# The flux qubit revisited to enhance coherence and reproducibility

**DOI:** 10.1038/ncomms12964

**Published:** 2016-11-03

**Authors:** Fei Yan, Simon Gustavsson, Archana Kamal, Jeffrey Birenbaum, Adam P Sears, David Hover, Ted J. Gudmundsen, Danna Rosenberg, Gabriel Samach, S Weber, Jonilyn L. Yoder, Terry P. Orlando, John Clarke, Andrew J. Kerman, William D. Oliver

**Affiliations:** 1 Research Laboratory for Electronics, Massachusetts Institute of Technology, Cambridge, Massachusetts 02139, USA; 2 Department of Physics, University of California, Berkeley, California 94720-7300, USA; 3 MIT Lincoln Laboratory, Quantum Information and Integrated Nanosystems Group, 244 Wood Street, Lexington, Massachusetts 02420, USA; 4 Department of Electrical Engineering and Computer Science, Massachusetts Institute of Technology, Cambridge, Massachusetts 02139, USA; 5 Department of Physics, Massachusetts Institute of Technology, Cambridge, Massachusetts 02139, USA

## Abstract

The scalable application of quantum information science will stand on reproducible and controllable high-coherence quantum bits (qubits). Here, we revisit the design and fabrication of the superconducting flux qubit, achieving a planar device with broad-frequency tunability, strong anharmonicity, high reproducibility and relaxation times in excess of 40 μs at its flux-insensitive point. Qubit relaxation times *T*_1_ across 22 qubits are consistently matched with a single model involving resonator loss, ohmic charge noise and *1/f*-flux noise, a noise source previously considered primarily in the context of dephasing. We furthermore demonstrate that qubit dephasing at the flux-insensitive point is dominated by residual thermal-photons in the readout resonator. The resulting photon shot noise is mitigated using a dynamical decoupling protocol, resulting in *T*_2_≈85 μs, approximately the 2*T*_1_ limit. In addition to realizing an improved flux qubit, our results uniquely identify photon shot noise as limiting *T*_2_ in contemporary qubits based on transverse qubit–resonator interaction.

## Abstract (web summary)

Scalable quantum information processing requires controllable high-coherence qubits. Here, the authors present superconducting flux qubits with broad frequency tunability, strong anharmonicity and high reproducibility, identifying photon shot noise as the main source of dephasing for further improvements.

Over the past 15 years, superconducting qubits have achieved a remarkable five-order-of-magnitude increase in their fundamental coherence metrics, including the energy-decay time *T*_1_ , the Ramsey free-induction decay time *T*_2_* , and the refocused Hahn-echo decay time *T*_2E_. This spectacular trajectory is traceable to two general strategies that improve performance: (1) reducing the level of noise in the qubit environment through materials and fabrication improvements, and (2) reducing the qubit sensitivity to that noise through design advancements^1^.

The charge qubit evolution is a quintessential example^2^. Early demonstrations (Cooper-pair box) exhibited nanosecond-scale coherence times^3^. Since then, operation at noise-insensitive bias points (quantronium)^4^, the introduction of capacitive shunting (transmon)^5^, the use of two-dimensional^6^ and three-dimensional (3D)^7^ resonators to modify the qubit electromagnetic environment, the development of high-Q capacitor materials and fabrication techniques^8^,^9^, and the introduction of alternative capacitor geometries (Xmon)^10^ have incrementally and collectively raised coherence times to the 10–100 μs range^10^,^11^ and beyond^12^,^13^. In addition, the capacitive shunt has generally improved device-to-device reproducibility. The trade-off, however, is a significant reduction in the charge qubit intrinsic anharmonicity (that is, the difference in transition frequencies *f*_01_ and *f*_12_ between qubit states 0, 1 and 1, 2) to 200–300 MHz for contemporary transmons, complicating high-fidelity control and exacerbating frequency crowding in multi-qubit systems^14^.

In contrast, the performance of the persistent-current flux qubit^15^,^16^ has progressed more slowly over the past decade. Device asymmetry was identified early on to limit flux qubit coherence^17^ and, since 2005, symmetric designs have generally achieved 0.5–5 μs (refs 18, 19) with a singular report of *T*_2E_=23 μs ≈2*T*_1_ (ref. 20). Despite respectable performance for individual flux qubits, however, device-to-device reproducibility has remained poor. An early attempt at capacitive shunting^21^ improved reproducibility, but coherence remained limited to 1–6 μs (refs 22, 23). Recently, flux qubits embedded in 3D (ref. 24) and coplanar^25^ resonators exhibited more reproducible and generally improved relaxation and coherence times: *T*_1_=6–20 μs, *T*_2_*=2–8 μs. Nonetheless, further improvements in these times and in reproducibility are necessary if the flux qubit is to be a competitive option for quantum information applications.

In this context we revisit the design and fabrication of the flux qubit. Our implementation, a capacitively shunted (C-shunt) flux qubit^21^ coupled capacitively to a planar transmission-line resonator, exhibits significantly enhanced coherence and reproducibility, while retaining an anharmonicity varying from 500–910 MHz in the four devices with the highest relaxation times. We present a systematic study of 22 qubits of widely varying design parameters—shunt capacitances *C*_sh_=9–51 fF and circulating currents *I*_p_=44–275 nA—with lifetimes at the flux-insensitive bias point ranging from *T*_1_<1 μs (small *C*_sh_, large *I*_p_) to *T*_1_=55 μs (large *C*_sh_, small *I*_p_). Over this entire range, the measured *T*_1_ values are consistent with a single model comprising ohmic charge noise, 1/*f-*flux noise, and Purcell-enhanced emission into the readout resonator. We furthermore investigated and identified quasiparticles as a likely source of observed *T*_1_ temporal variation. For the highest coherence devices, the Hahn-echo decay time *T*_2E_=40 μs<2*T*_1_ does not reach the 2*T*_1_ limit, as is also often observed with transmons coupled transversally to resonators^7^,^10^,^26^. We demonstrate that this is due to dephasing caused by the shot noise of residual photons in the resonator (mean photon number 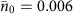), observing a lorentzian noise spectrum with a cutoff frequency consistent with the resonator decay rate. We then use Carr–Purcell–Meiboom–Gill (CPMG) dynamical decoupling to recover *T*_2CPMG_≈2*T*_1_ in a manner consistent with the measured noise spectrum.

## Results

### C-shunt flux qubit

**Figure 1:**
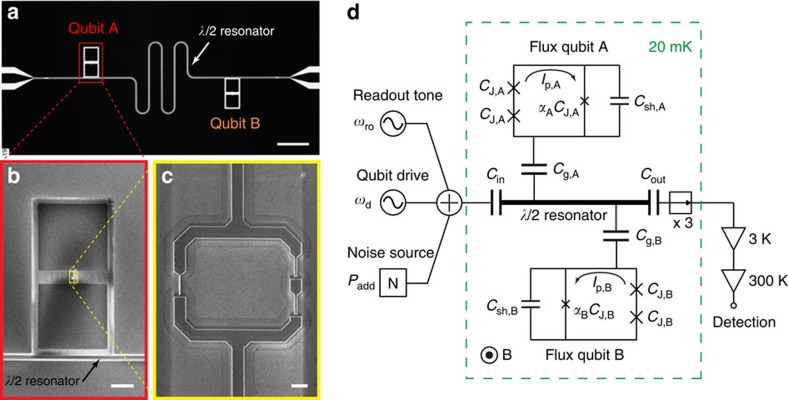
C-shunt flux qubit. (**a**) Optical micrograph of the 2.5 × 5.0 mm^2^ chip, aluminium (black) on sapphire substrate (white, where the aluminium has been etched away), featuring two qubits (A and B) and a *λ*/2 coplanar waveguide resonator (*ω*_r_/2*π*=8.27 GHz). Scale bar, 0.5 mm. (**b**) SEM image of the shunt capacitor (*C*_sh,A_=51 fF) for qubit A. Each square plate of the capacitor is 200 × 200 μm^2^. The lower plate capacitively couples the qubit to the *λ*/2 resonator. Scale bar, 50 μm. (**c**) Magnified view of the shadow-evaporated qubit loop and its three Josephson junctions. The left junction area is smaller by a factor *α*_A_=0.42. Scale bar, 1 μm. (**d**) Device and measurement schematic. Experiments are performed in a dilution refrigerator at 20 mK. A global magnetic field *B* provides a magnetic flux bias Φ_b_ to each qubit. A qubit drive tone (*ω*_d_), readout tone (*ω*_ro_) and externally generated noise (*P*_add_, see Figs 5 and 6) enter the *λ*/2 resonator defined by capacitances *C*_in_ and *C*_out_. The resonator is capacitively coupled (*C*_g,A/B_) to qubits A and B. The qubit junctions (‘x') have internal capacitance, *C*_J,A/B_ and *α*_A/B_*C*_J,A/B_, and are externally shunted by capacitance *C*_sh,A/B_. Each qubit loop supports a circulating persistent current *I*_p,A/B_. Readout signals at the resonator output pass three isolators (‘→'), are amplified at cryogenic and room temperatures, and subsequently detected. See supplementary online material for more information.

Our circuits each contain two C-shunt flux qubits—with different frequencies—placed at opposite ends of a half-wavelength superconducting coplanar waveguide resonator (Fig. 1a). The resonator, ground plane and capacitors (Fig. 1b) were patterned from MBE-grown aluminium deposited on an annealed sapphire substrate^8^ (Supplementary Note 1). We used both square capacitors (Fig. 1b) and interdigital capacitors (IDCs, not shown) coupled capacitively to the centre trace of the coplanar waveguide resonator to enable qubit control and readout. In a second fabrication step, the qubit loop and its three Josephson junctions (Fig. 1c) were deposited using double-angle, electron-beam, shadow evaporation of aluminium. One junction is smaller in area (critical current) by a factor *α*, and each of its leads contacts one electrode of the shunt capacitor. An equivalent circuit is illustrated in Fig. 1d (Supplementary Note 2).

Varying the qubit design enables us to explore a range of qubit susceptibilities to flux and charge noise with impact on both *T*_1_ and *T*_2_ (ref. 21). Compared with the conventional persistent-current flux qubit^15^,^16^, our best C-shunt flux qubits have two key design enhancements. First, a smaller circulating current—achieved by reducing the area and critical current density of the Josephson junctions (Fig. 1c)—reduces the qubit sensitivity to flux noise, a dominant source of decoherence in flux qubits. Second, a larger effective junction capacitance—achieved by capacitively shunting the small junction (Fig. 1b)—reduces the qubit sensitivity to charge noise, and improves device reproducibility by reducing the impact of both junction fabrication variation and unwanted stray capacitance. Furthermore, the use of high-quality fabrication techniques and physically large shunt capacitors reduces the density and electric participation of defects at the various metal and substrate interfaces^1^.

The system is operated in the dispersive regime of circuit quantum electrodynamics and is described by the approximate Hamiltonian^27^


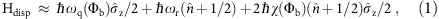


where, the three terms are respectively the qubit (represented as a two-level system), resonator and qubit–resonator interaction Hamiltonians, 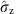 is the Pauli operator defined by the qubit energy eigenbasis, *ω*_r_ is the resonator angular frequency and 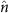 is the resonator photon-number operator. The qubit angular frequency *ω*_q_(Φ_b_) is set by the magnetic flux bias Φ_b_, measured relative to an applied flux (*m*+1/2)Φ_0_ where *m* is an integer and Φ_0_ is the superconducting flux quantum, and attains its minimum value *ω*_q_(0)≡Δ at the flux-insensitive point Φ_b_=0. The quantity *χ*(Φ_b_) is the qubit-state-dependent dispersive shift of the resonator frequency, which is used for qubit readout. In the Supplementary Notes 3–5, we discuss further the two-level system approximation for the C-shunt flux qubit, an approximate analytic treatment which goes beyond equation (1), and the numerical simulation of the full qubit–resonator Hamiltonian used to make quantitative comparisons with our data.

### *T*_1_ relaxation and noise modelling

**Figure 2:**
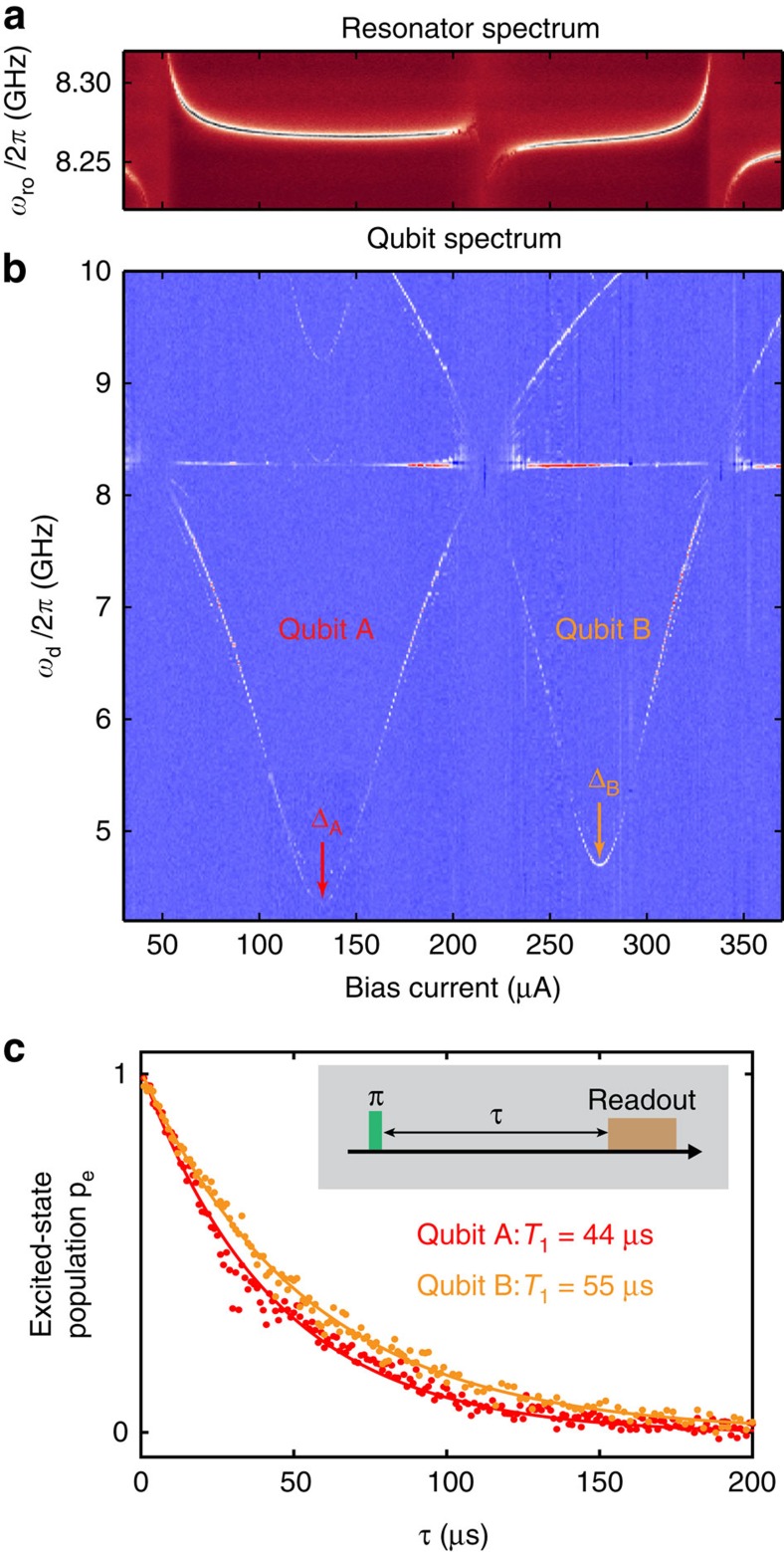
Spectroscopy and *T*_1_ of two capacitively shunted flux qubits. (**a**,**b**) Resonator and qubit spectra versus bias current used to induce the global magnetic field *B*. The qubit transition frequencies *ω*_q_/2*π* have minima Δ_A_/2*π*=4.36 GHz and Δ_B_/2*π*=4.70 GHz at the qubit flux-insensitive points, which are intentionally offset in bias current (magnetic flux) by using different qubit-loop areas. (**c**) Energy-decay functions of qubits A and B measured at their respective degeneracy points using the inversion-recovery pulse sequence (inset). Solid lines are exponential fits with decay constant *T*_1_.

We begin by presenting the *T*_1_ characterization protocol for the device in Fig. 1. We first identify the resonator transmission spectrum (Fig. 2a) by scanning the readout-pulse frequency *ω*_ro_ about the bare resonator frequency *ω*_r_/2*π*≈8.27 GHz. Using standard circuit quantum electrodynamics readout, qubit-state discrimination is achieved by monitoring the qubit-state-dependent transmission through the resonator^27^. Next, we add a qubit driving pulse of sufficient duration to saturate the ground-to-excited-state transition and sweep the pulse frequency *ω*_d_ (Fig. 2b). The resulting spectra for qubits A and B (Fig. 1a) exhibit minima Δ_A_/2*π*≈4.4 GHz and Δ_B_/2*π*≈4.7 GHz at the qubit flux-insensitive points and increase with magnetic flux (bias current) away from these points. Finally, using a single *π*-pulse to invert the qubit population, we measure the *T*_1_ relaxation of qubit A (*T*_1_=44 μs) and qubit B (*T*_1_=55 μs) at their flux-insensitive points (Fig. 2c). High-power spectroscopy (see Supplementary Note 6) reveals transitions among the first four qubit energy levels that are well matched by simulation, and identifies anharmonicities of 500 MHz in the two measured devices.

Using this protocol, we investigated 22 C-shunt flux qubits from five wafers (fabrication runs), spanning a range of capacitance values (*C*_sh_=9–51 fF) and qubit persistent currents (*I*_p_=44–275 nA) and featuring two capacitor geometries (interdigital and square). The junction critical currents were adjusted to maintain Δ/2*π*≈0.5–5 GHz (see Supplementary Note 7).

The data were analysed using simulations of the full system Hamiltonian and a Fermi's golden rule expression for the exited state decay rate^21^,


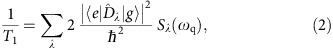


where |*g*〉(|*e*〉) indicates the qubit ground (excited) states, and the sum is over four decay mechanisms: flux noise in the qubit loop, charge noise on the superconducting islands, Purcell-enhanced emission to the resonator mode, and inelastic quasiparticle tunnelling through each of the three junctions. The operator 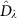 is a transition dipole moment, and *S*_*λ*_(*ω*_q_) is the symmetrized noise power spectral density for the fluctuations which couple to it. For example, 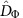 is a loop current operator for flux noise *S*_Φ_(*ω*), and 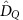 is an island voltage operator for charge noise *S*_*Q*_(*ω*) (Supplementary Notes 8 and 9).

We considered both *S*_*λ*_(*ω*)∝1/*ω*^*γ*^ (inverse-frequency noise) and *S*_*λ*_(*ω*)∝*ω* (ohmic noise)—the two archetypal functional forms of noise in superconducting qubits^20^,^28^,^29^,^30^,^31^,^32^,^33^—for our magnetic flux and charge noise models, and used the frequency dependence of *T*_1_ for specifically designed devices to distinguish between them. While the following results are presented using symmetrized power spectral densities, we are careful to account for the distinction between classical and quantum noise processes in making this presentation (Supplementary Note 9).

**Figure 3:**
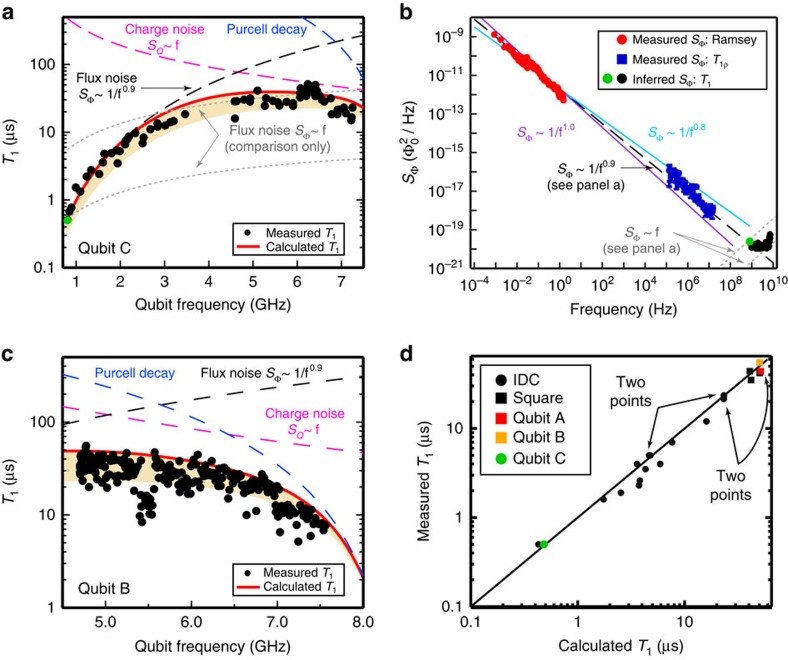
T_1_ variation with qubit frequency and noise modelling. (**a**) Energy-relaxation time *T*_1_ versus qubit frequency for Qubit C (*C*_sh,C_=9 fF, *I*_p,C_=275 nA, Δ_c_/2*π*=0.82 GHz) plotted with simulated *T*_1_ values for individual (dashed lines) and aggregate (solid line) charge, flux and Purcell noise mechanisms. Absence of data around 4 GHz is related to an ancillary qubit level crossing the readout resonator, prohibiting qubit readout and is not a systematic issue. Qubit C is limited by flux noise below about 4.5 GHz. For comparison, the functional form for ohmic flux noise (grey dotted line) is incompatible with the data below 3 GHz; above 3 GHz, its role cannot be readily distinguished from charge noise (see text). Shaded region indicates the range of predicted *T*_1_ in the presence of 0–1.0 quasiparticles. (**b**) Flux noise spectroscopy performed on Qubit B using Ramsey interferometry (red) and T_1*ρ*_ spin-locking (blue) to determine parameters *A*_Φ_^2^=(1.4 μΦ_0_)^2^/Hz and *γ* =0.9 for the inverse-frequency flux noise (black dashed line) for qubit C (**a**). Green and black dots: inferred ohmic flux noise *S*_Φ_ based on measured *T*_1_ in **a**. (**c**) Energy-relaxation time *T*_1_ versus qubit frequency for qubit B (*C*_sh,B_=51 fF, *I*_p,B_=49 nA, 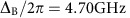). *T*_1_ is sensitive predominantly to ohmic charge noise within 5–6.5 GHz range. Scatter in *T*_1_ is attributed to quasiparticle fluctuations. Cluster of lower *T*_1_ values near 5.5 GHz is due to interaction with the *f*_12_ transition. Shaded region indicates the range of predicted *T*_1_ in the presence of 0–1.0 quasiparticles. (**d**) *T*_1_ values for 22 qubits with widely varying design parameters, measured at their degeneracy points and plotted against predicted *T*_1_ values (dashed line) determined from numerical simulations using a single model with fixed noise levels (see main text). Practically indistinguishable data points (eight in total) are indicated with arrows.

For example, in Fig. 3a, Qubit C (*C*_sh_=9 fF) has a large persistent current (*I*_p_=275 nA) and a small qubit frequency (Δ_c_*/*2*π*=0.82 GHz), making it highly sensitive to flux noise. Consequently, the measured *T*_1_ is predominantly limited by flux noise over a wide frequency range. This *T*_1_-trend constrains the flux noise model to the form *S*_Φ_*(ω*)≡*A*_Φ_^2^(2*π* × 1 Hz*/ω*)^γ^ over the range 0.82–3 GHz (black dashed line, Fig. 3a). For comparison, the functional form for ohmic flux noise (grey dashed line), scaled to match *T*_1_ at Δ_c_*/*2*π*=0.82 GHz (green dot), is clearly inconsistent with all other data over this frequency range. The noise parameters *A*_Φ_^2^=(1.4 μΦ_0_)^2^/Hz and *γ*=0.9 used to match the data in Fig. 3a are derived from independent measurements—Ramsey interferometry^31^ and *T*_1ρ_ noise spectroscopy^32^ (Supplementary Note 10)—made at much lower frequencies in the context of classical noise related to qubit dephasing (Fig. 3b). These values are commensurate with earlier work on qubits^20^,^31^,^32^,^33^ and d.c. Superconducting QUantum Interference Devices (SQUIDs)^34^. The consistency between the magnitude and slope of the flux noise power spectra, spanning more than twelve decades in frequency—millihertz to gigahertz—is remarkable, made even more so by the fact that the data in Fig. 3b were measured with a different device (qubit B, Fig. 3c).

In contrast, Qubit B (*C*_sh_=51 fF) has a much smaller persistent current (*I*_p_=49 nA) and larger qubit frequency (Δ_B_/2*π*=4.7 GHz). Its value of *T*_1_ is most strongly influencedby charge noise (magenta dashed line, Fig. 3c) in the 5.0–6.5 GHz range, consistent with an ohmic charge noise model of the form *S*_*Q*_(*ω*)≡*A*_*Q*_^2^*ω*/(2*π* × 1 GHz) with the parameter *A*_*Q*_^2^=(5.2 × 10^−9^*e*)^2^/Hz adjusted to match the data. In addition to flux and charge noise, the predicted value of *T*_1_ due to Purcell loss (light blue dashed line) is also included in Fig. 3a,c and involves no free parameters (see Supplementary Note 8). The resulting net value of *T*_1_ due to all three mechanisms (inverse-frequency flux noise, ohmic charge noise and Purcell loss) is indicated with a red solid line and is in relatively good agreement with the ceiling of measured *T*_1_ values. As we describe below, quasiparticles are responsible for reducing the *T*_1_ below this ceiling.

Using these models, Fig. 3d shows a comparison of the measured and predicted *T*_1_ values for all 22 qubits. The flux noise model (from Fig. 3a,b) is applied to all qubits, and the Purcell loss is included with no free parameters. For the charge noise model, to achieve agreement across all devices, it was necessary to use *A*_*Q*,SQ_^2^=(5.2 × 10^−9^*e*)^2^/Hz for square capacitors (from Fig. 3b) and *A*_*Q*,IDC_^2^=(11.0 × 10^−9^*e*)^2^/Hz for IDCs, presumably reflecting the larger electric participation of the surface and interface defects for the IDC geometry^1^. The agreement is noteworthy, given that these qubits span a wide range of designs across five fabrication runs (see Supplementary Note 7).

We note that inverse-frequency charge noise was incompatible with these data over the entire frequency range investigated (not shown), implying that the cross-over between inverse-frequency and ohmic charge noise occurred at a frequency below 0.82 GHz. However, while ohmic flux noise *S*_Φ_(*ω*)∝*ω* was inconsistent with *T*_1_ over the frequency range 0.82–3 GHz, its functional form is plausibly consistent with data above 3 GHz when appropriately scaled (upper dashed grey line, Fig. 3a) and, therefore, cannot be conclusively distinguished from ohmic charge noise. Although the best agreement across all 22 qubits (Fig. 3d) did not require ohmic flux noise, we could not rule out its presence in the 3–7 GHz range. In Supplementary Note 11, we compare models that use ohmic charge noise (as in Fig. 3) and ohmic flux noise. Differentiating between such charge and flux noise at higher frequencies will be the subject of future work. Indeed, for both ohmic flux noise *S*_Φ_(*ω*)∝*ω* and inverse-frequency charge noise *S*_*Q*_(*ω*)∝1/*ω*, it is certainly possible (even expected) that the former (latter) dominates the flux (charge) noise at sufficiently higher (lower) frequencies.

**Figure 4:**
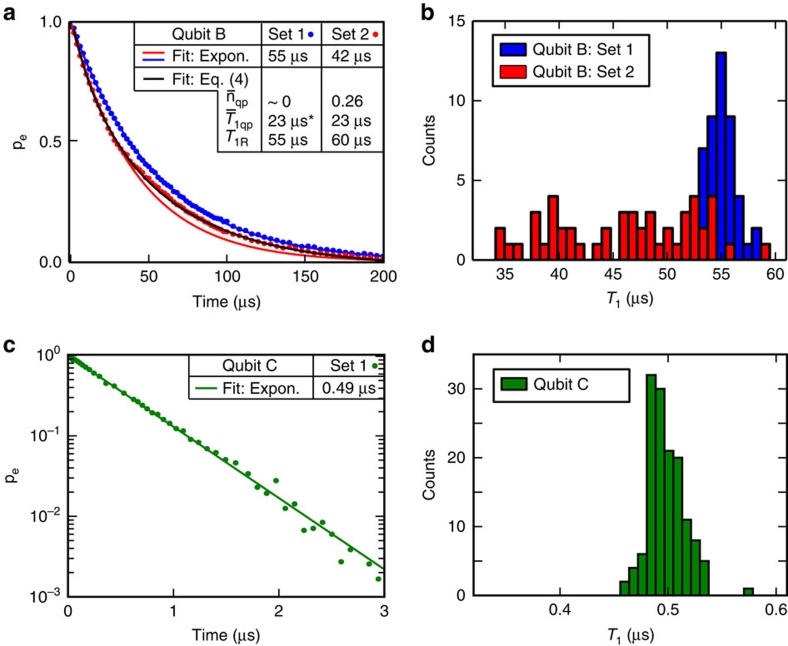
T_1_ temporal variation and quasiparticles. (**a**) Energy-relaxation measurements (set 1 and set 2) at *ω*_q_=Δ_B_ for qubit B. Each set comprises the average of 50 individual decay traces acquired sequentially in 4-min intervals. Set 1 exhibits purely exponential decay with *T*_1_=55 μs, whereas set 2 (acquired 17 h after set 1) exhibits a non-exponential decay function. The black line is a fit to equation (3) assuming the non-exponential decay is due to quasiparticle fluctuations (see text). Inset: tabulation of the values obtained from fitting functions. The ‘*' indicates an assumed value from set 2 (not a fit value). (**b**) Histograms of *T*_1_ values obtained by exponential fits of the individual traces forming the two data sets in **a**. For set 2, the fitting is restricted to the first 40 μs to capture primarily the fast initial decay. (**c**) Energy-relaxation measurements at *ω*_q_=Δ_C_ for qubit C. The exponential decay function is manifest as a linear fit on the log plot with time constant *T*_1_=0.49 μs. (**d**) Histograms of *T*_1_ values obtained from repeated measurements of qubit C. Both the exponential decay function (**c**) and the consistently tight *T*_1_ distribution (**d**) indicate a relative insensitivity to quasiparticle number fluctuations.

The measured data for qubit B (Fig. 3c) exhibit fluctuations in the range *T*_1_=20–60 μs for qubit frequencies ω_q_/2*π=*4.7–6.5 GHz. To investigate their temporal nature, we measured *T*_1_ repeatedly at the qubit flux-insensitive point 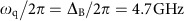 over a 10-h period and collected the data into sets of 50 individual decay traces. Fig. 4a,b show the results of two such experiments, with set 2 being taken ∼17 h after set 1. The average of all traces from set 1 exhibits a purely exponential decay, whereas the corresponding average for set 2 exhibits a faster short-time decay and clear non-exponential behaviour (Fig. 4a). Histograms of the *T*_1_ values for individual traces exhibit a tight, Gaussian-shaped distribution centred at 55 μs for set 1 and a broader, quasi-uniform distribution centred near 45 μs for set 2. Over the course of several weeks, we observed transitions between these two characteristic modes of behaviour every few days for this device^35^.

We attribute the temporal fluctuations and non-exponential decay function to excess quasiparticles—above the thermal equilibrium distribution—near the qubit junctions^36^,^37^,^38^,^39^. Following ref. 40, we define 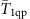 as the average relaxation time associated with a single quasiparticle and take the quasiparticle number *n*_qp_ to be Poisson-distributed with mean value 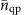. This results in a qubit polarization decay function,


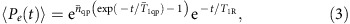


where *T*_1R_ captures the residual exponential decay time in the absence of quasiparticles 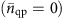. The non-exponential decay function observed for set 2 is well described by equation (3) (black line in Fig. 4a) with fitting parameters 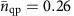, 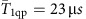 and *T*_1R_=60 μs.

We use a quantum treatment of quasiparticle tunnelling to model the impact of single quasiparticles on the *T*_1_ of qubit B (Supplementary Note 8). Using a quasiparticle density *x*_qp_=4 × 10^−7^ (per superconducting electron), the calculated 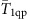 recovers the fitted value 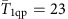
μs at the flux-insensitive point. Both 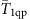 and *x*_qp_ are comparable to the quasiparticle-induced relaxation rates and quasiparticle density reported for similar devices^24^,^41^. The shaded region in Fig. 3a,c indicates the range of predicted *T*_1_ in the presence of 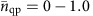 quasiparticle. Most *T*_1_ data lie within this region, supporting the hypothesis that their scatter (particularly for qubit B in Fig. 3c) and the observed temporal *T*_1_ variation (Fig. 4b) arise from the common mechanism of quasiparticle tunnelling. In addition, the residual relaxation time *T*_1R_ for set 2 is similar to the exponential time constant obtained for set 1, indicating an underlying consistency in the noise models between the two data sets in the absence of quasiparticles. Unlike qubit B, qubit C consistently exhibited an exponential decay function (Fig. 4c) with little temporal variation (Figs 3a and 4d), indicating that quasiparticles did not strongly influence this device.

The results of Figs 3 and 4 demonstrate clearly that 1/*f*-type flux noise is the dominant source of qubit relaxation for frequencies below 3 GHz. To further strengthen this claim, it is instructive to compare relaxation times for qubits with similar frequencies and shunting capacitances, but where the persistent current (and thereby the sensitivity to flux noise) differs. We find that by reducing *I*_p_ from 170 nA to 60 nA, we improve the measured *T*_1_ from 2.3 to 12 μs (see qubits 11 and 13 in Supplementary Table 1 in Supplementary Note 7).

### Pure dephasing and thermal-photon noise

We now address the transverse relaxation time *T*_2_ and our ability to refocus coherent dephasing errors. Efficient refocusing implies that *T*_2_ is limited entirely by *T*_1_, since 1/*T*_2_=1/2*T*_1_+1/*T*_*ϕ*_, where *T*_*ϕ*_ is the dephasing time. Generally, *T*_2_ is maximal at the flux-insensitive point for conventional flux qubits^18^,^19^,^20^, and the device reported in ref. 20 was efficiently refocused with a single echo pulse (*T*_2E_=23 μs≈2*T*_1_). In the current work, however, a single refocusing pulse is no longer completely efficient (*T*_2*E*_<2*T*_1_). This suggests that an additional, higher-frequency noise channel has been introduced. Unlike the device in ref. 20, which was coupled to a d.c. SQUID for readout, our C-shunt flux qubits are transversally coupled to a resonator (Fig. 1). Such inefficient refocusing is also reported for transmons similarly coupled to resonators^7^,^10^,^26^.

As we show below, the main source of dephasing in C-shunt flux qubits biased at their flux-insensitive point is photon-number fluctuations (shot noise) in the resonator, which vary the qubit frequency via the a.c. Stark effect (as in the transmon case^26^,^27^). Given a small thermal-photon population 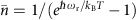
<<1 in the resonator (see Supplementary Note 12), the photon-induced frequency shift Δ_Stark_^th^ and dephasing rate 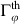 of the qubit are^42^


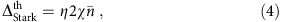



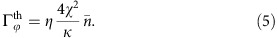


**Figure 5:**
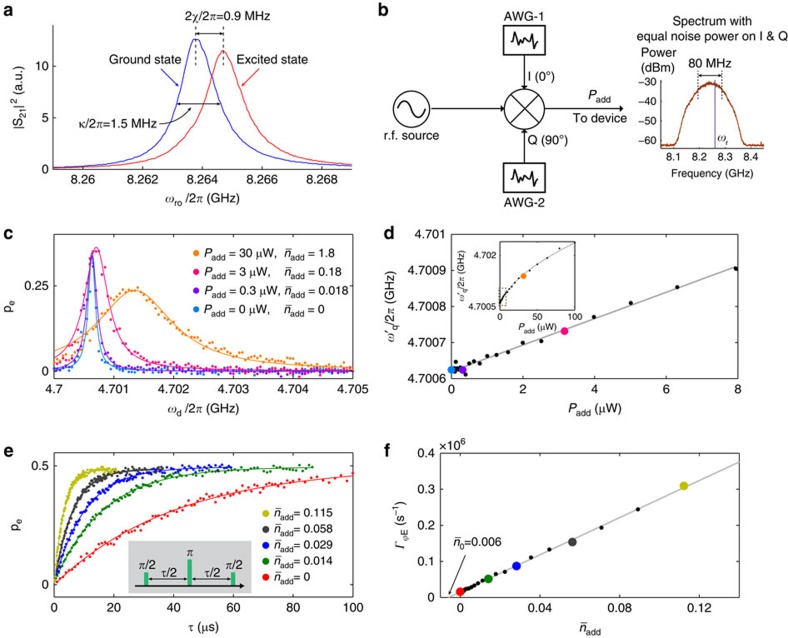
Calibration of engineered noise. (**a**) Resonator transmission spectra measured with the qubit prepared in the ground and excited states. In contrast to typical transmon qubits with *ω*_q_<*ω*_r_, an excited-state C-shunt flux qubit shifts the resonator to higher frequencies because of interactions with higher-level qubit transitions. (**b**) Engineered thermal-photon noise source. A coherent tone near the resonator frequency is mixed with white-noise of nominally equal power from two independent arbitrary waveform generators (AWGs) applied to the in-phase (I) and quadrature (Q) ports of the I/Q mixer. The AWG noise bandwidth (80 MHz) is much greater than the cavity linewidth, creating effectively a thermal-photon noise source with power *P*_add_. (**c**) Qubit spectral line shape (dots) and lorentzian fits (solid lines) for various added noise powers *P*_add_. The equivalent photon population 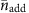 added to the resonator is derived from **d**. The blue trace corresponds to no added noise from the source in **b**. (**d**) Stark-shifted qubit frequency versus applied noise power (dots). Coloured dots correspond to traces in **c**. Combining the linear fit (solid line) with the first-order dependence of Stark shift on photon population (equation (4)) yields the power-per-added-photon 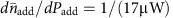 in the low-power limit. Inset: wider range of applied noise powers; dashed box indicates the range in the main panel. At large photon populations 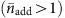 the frequency shift becomes nonlinear, following equation 43 in ref. 40 (solid line). (**e**) Spin-echo decay (dots) with exponential fit (solid lines) for several values of added photons. Inset: spin-echo pulse sequence. (**f**) Spin-echo pure-dephasing rate (echo decay rate without the *T*_1_ contribution) plotted versus injected photon population (dots). The linear fit (solid line) has slope 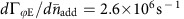, in agreement with the value of 2.5 × 10^6^ s^−1^ calculated from equation (5). The intercept indicates a residual photon population 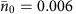 in the resonator.

The factor *η*=*κ*^2^/(*κ*^2^+4*χ*^2^) effectively scales the photon population seen by the qubit due to the interplay between the qubit-induced dispersive shift of the resonator frequency *χ* and the resonator decay rate *κ*. Both the strong dispersive (2*χ*>>*κ*) and weak dispersive (2*χ*<<*κ*) regimes have been previously addressed^26^,^43^,^44^. Here, we use qubit B to focus primarily on the intermediate dispersive regime (2*χ*/2*π*=0.9 MHz, *κ*/2*π*=1.5 MHz, see Fig. 5a) relevant for high-fidelity qubit readout^45^.

We begin by intentionally injecting additional thermal-photons 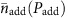 into the resonator from an external noise generator with power *P*_add_ (Fig. 5b and Supplementary Note 2). In the small-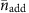 limit, the measured qubit spectrum exhibits a linear relationship between the effective qubit frequency 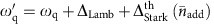 and the generator power *P*_add_ (Fig. 5c,d). For completeness, we have included the Lamb shift Δ_Lamb_, a fixed frequency offset due to the resonator zero-point energy. Combining the extracted slope with equation (4), we calibrate the dependence of the added-photon population 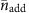 (in the resonator) on the generator power *P*_add_.

Next, we measure the Hahn-echo dephasing rate for several photon populations using the calibrated 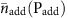. All echo traces (Fig. 5e) feature exponential decay rates Γ_2E_=1/*T*_2E_, indicating little (if any) impact from 1/*f* noise (charge, flux and so on) and consistent with photon shot noise featuring a short correlation time 1/*κ<<T*_2E_. The extracted pure dephasing rate 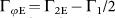 scales linearly with photon population 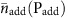 (Fig. 5f). The extracted slope agrees with equation (5) to within 5%. The non-zero dephasing rate at 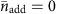 corresponds to a residual photon population 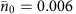, equivalent to an effective temperature *T*_eff_=80 mK. By comparison, the qubit effective temperature determined from its first excited-state population is 35 mK (ref. 13).

**Figure 6:**
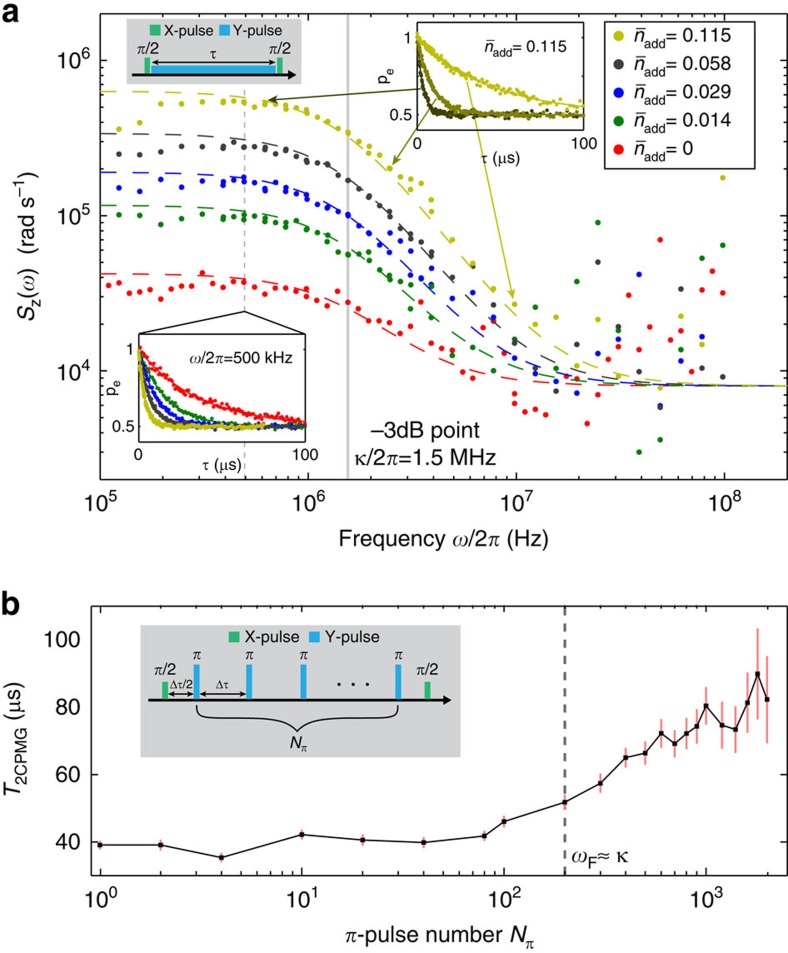
PSD of photon fluctuations in the resonator. (**a**) Noise power spectral densities (PSDs) extracted from spin-locking (*T*_1*ρ*_) relaxation experiments (Inset, top-left: *T*_1*ρ*_ pulse sequence) measured for different locking (Rabi) frequencies (0.1–100 MHz) and added noise photons 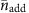. Coloured dashed lines indicate expected lorentzian noise spectra (see equation (6)) assuming a constant white-noise offset. The vertical grey line indicates the 3-dB point of the lorentzians, coinciding with the resonator decay rate *κ*/2*π*=1.5 MHz. Inset, bottom-left: *T*_1*ρ*_ decay traces for different photon populations at fixed locking (Rabi) frequency *ω*/2*π*=Ω_R_/2*π*=500 kHz. Inset, top-right: *T*_1*ρ*_ decay traces at different locking (Rabi) frequencies for *n*_add_=0.115. (**b**) Decay times *T*_2CPMG_ for the CPMG sequence measured versus number of *π*-pulses, *N*_*π*_, with *n*_add_=0 (no added noise photons). The CPMG pulse sequence (inset) acts as a bandpass noise filter centred at a frequency proportional to *N*_*π*_ through the pulse spacing Δ*τ* (see main text). At *N*_*π*_=200, the filter frequency approximately equals the cavity decay rate (dashed line). For *N*_*π*_<200, the filter samples the flat low-frequency portion of the lorentzian PSD, yielding a constant decay time *T*_2CPMG_≈40 μs. For *N*_*π*_>200, the filter traverses the roll-off region of the lorentzian. As the sampled noise decreases, the decay times increase, approaching the limit set by energy relaxation (*T*_2CPMG_≈2*T*_1_) for *N*_*π*_>1,000. Error bars indicate 95% confidence intervals on the fitting algorithm used to extract these data.

To confirm that the noise arises from residual thermal-photons, we directly measure the noise power spectral density (PSD) using the *T*_1*ρ*_ (spin-locking) method^32^. This method (inset Fig. 6a) collinearly drives the qubit along the *y*-axis with a long *Y* pulse, which ‘locks' the qubit state in the rotating frame. Measuring the qubit relaxation rate in the rotating frame, Γ_1*ρ*_(Ω_Rabi_)=*S*_z_(Ω_Rabi_)/2+Γ_1_/2, effectively samples the noise PSD *S*_z_(*ω*) seen by the qubit at the locking (Rabi) frequency Ω_Rabi_ (see Supplementary Note 10). By varying the locking drive amplitude, which is proportional to Ω_Rabi_, we sample the noise spectrum over the range *ω*/2*π*=0.1–100 MHz (Fig. 6a). Below 10 MHz, the resolved noise spectra for all 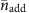 (including 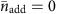) have similar shapes: flat (white) at low frequencies with a 3-dB high-frequency cutoff at the resonator decay rate *ω*=*κ*. This form is consistent with the expected lorenzian PSD for thermal-photons in a resonator as seen by the qubit (see Supplementary Note 10),


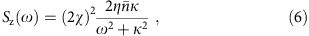


which includes the dispersive coupling *χ* and the filtering factor *η* [see equations (4 and 5)]. Equation (6) agrees with the measured PSDs for all photon populations 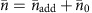, with the residual photon number 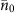 extracted from equation (6). This agreement eliminates the driving or readout field as the source of the residual photons, because such coherent-state photons follow Poisson statistics with a resulting cutoff frequency *κ*/2 (half the observed value)^46^,^47^.

Finally, we apply dynamical decoupling techniques to validate the functional form of the measured noise PSD and to recover *T*_2_≈2*T*_1_. We use the CPMG (inset Fig. 6b) pulse sequence, comprising a number *N*_*π*_ of equally spaced *π*-pulses. The application of *π*-pulses in the time domain can be viewed as a bandpass filter in the frequency domain which shapes the noise spectra seen by the qubit^21^,^48^,^49^,^50^. Since the filter passband is centred at a frequency inversely related to the temporal spacing Δ*τ* between adjacent pulses, increasing *N*_*π*_ for a fixed sequence length will shift this passband to higher frequencies (see Supplementary Note 13).

Figure 6b shows the measured CPMG decay time *T*_2CPMG_ versus *π*-pulse number *N*_*π*_ with no added noise 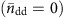. From *N*_*π*_=1 (Hahn-echo) to *N*_*π*_=100, the decay time *T*_2CPMG_ remains near 40 μs, consistent with the white-noise (flat) portion of the noise PSD in Fig. 6a. Above *N*_*π*_=100, the passband frequency traverses the cutoff region of the PSD and, as the integrated noise level decreases, *T*_2CPMG_ rises. For *N*_*π*_>1,000, the refocusing becomes efficient with 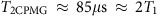. The close correspondence between the noise spectral density in Fig. 6a and the mitigation of that noise by CPMG in Fig. 6b strongly supports our methods and interpretations.

## Discussion

The C-shunt flux qubit is a planar device with broad-frequency tunability, relatively strong anharmonicity and high reproducibility, making it well suited to both gate-based quantum computing and quantum annealing. The anharmonicity can be significantly higher than that of transmon qubits, allowing for faster (even subnanosecond^51^,^52^) control pulses and reduced frequency crowding in multi-qubit systems. The addition of a high-quality-factor shunt capacitance to the flux qubit, together with a reduced qubit persistent current, has enabled us to achieve values of *T*_1_ as high as 55 μs at the qubit flux-insensitive point. We are able to account for measured *T*_1_ values across 22 qubits with a single model involving ohmic charge noise, 1/*f-*flux noise, and the Purcell effect, with temporal variation in *T*_1_ explained by quasiparticle tunnelling. On the basis of this model, we anticipate further design optimization leading to even higher coherence will be possible. Finally, we used spin-locking to directly measure the photon shot noise spectral density, and we verified its functional form using a CPMG pulse sequence to reach a *T*_2_ of 85 μs—limited by 2*T*_1_—at the flux-insensitive point. These measurements identify photon shot noise as the dominant source of the observed dephasing, and have direct implications for any qubit in which the readout involves its transverse coupling to a resonator.

The role of high-frequency 1/*f-*flux noise in qubit relaxation is intriguing. Our *T*_1_ data and their frequency dependence across 22 different qubits strongly support the conclusion that 1/*f*-flux noise contributes to qubit relaxation up to at least 3 GHz in our devices. Above 3 GHz, there is some ambiguity between ohmic flux and ohmic charge noise, and clarifying the roles of these respective noise sources is the subject of future work. A detailed understanding of such a broadband 1/*f*-flux noise mechanism and its transition from classical to quantum behaviour is of great practical interest and awaits theoretical explanation.

## Data availability

The data that support the findings of this study may be made available from the corresponding author upon request and with the permission of the US Government sponsors who funded the work.

## Additional information

**How to cite this article:** Yan, F. *et al*. The flux qubit revisited to enhance coherence and reproducibility. *Nat. Commun.* **7,** 12964 doi: 10.1038/ncomms12964 (2016).

## Supplementary Material

Supplementary Information Supplementary Figures 1-11, Supplementary Table 1, Supplementary Notes 1-13 and Supplementary References.
