# Supplementary material for: The flux qubit revisited to enhance coherence and reproducibility
Source: Nat Commun. 2016 Nov 3;7:12964. doi: 10.1038/ncomms12964 (PMC5097147; doi:10.1038/ncomms12964)
Supplement: Supplementary Information — Supplementary Figures 1-11, Supplementary Table 1, Supplementary Notes 1-13 and Supplementary References. [file ncomms12964-s1.pdf]

Supplementary Materials:

The Flux Qubit Revisited to Enhance Coherence and Reproducibility

F. Yan[1], S. Gustavsson[1], A. Kamal[1], J. Birenbaum[2,*], A. Sears[3], D. Hover[3],

T. Gudmundsen[3], D. Rosenberg[3], G. Samach[3], S. Weber[3], J. Yoder[3],

T.P. Orlando[1,4], John Clarke[2], A.J. Kerman[3], & W.D. Oliver[1,3,5]

[1]Research Laboratory of Electronics,
Massachusetts Institute of Technology, Cambridge, MA 02139, USA.
[2]Department of Physics, University of California, Berkeley, CA 94720-7300, USA.
[3]MIT Lincoln Laboratory, 244 Wood Street, Lexington, MA 02420, USA.
[4]Department of Electrical Engineering and Computer Science,
[5]Department of Physics,
Massachusetts Institute of Technology, Cambridge, MA 02139, USA.
[*]Current address: MIT Lincoln Laboratory; jeffrey.birenbaum@ll.mit.edu

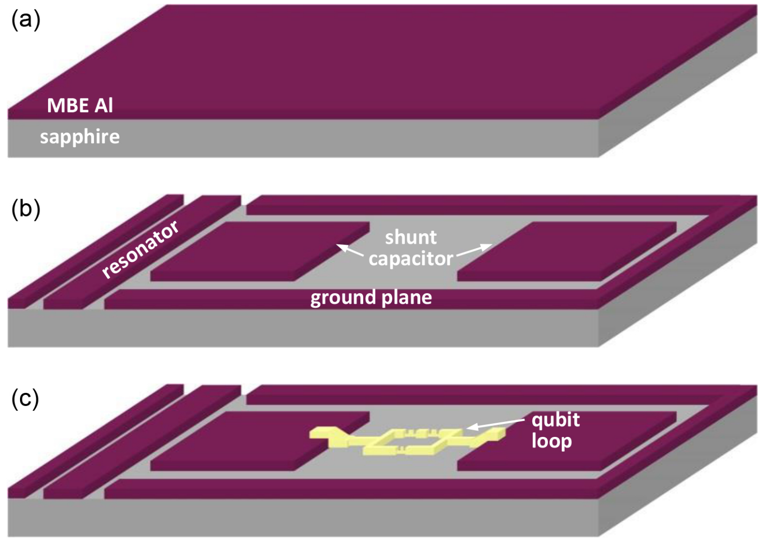

Supplementary Figure 1: **Process flow schematics of key steps for capacitively-shunted flux qubit fabrication. a**, Preparation of MBE aluminum (red) on outgassed C-plane sapphire substrates (gray). **b**, Patterning of the MBE aluminum into the shunt capacitor (representative square shunt capacitor geometry is shown), resonator center line, and surrounding ground plane. **c**, Patterning of the aluminum qubit loop (yellow), which contains three aluminum Josephson junctions. The loop contacts the shunt capacitor as illustrated.

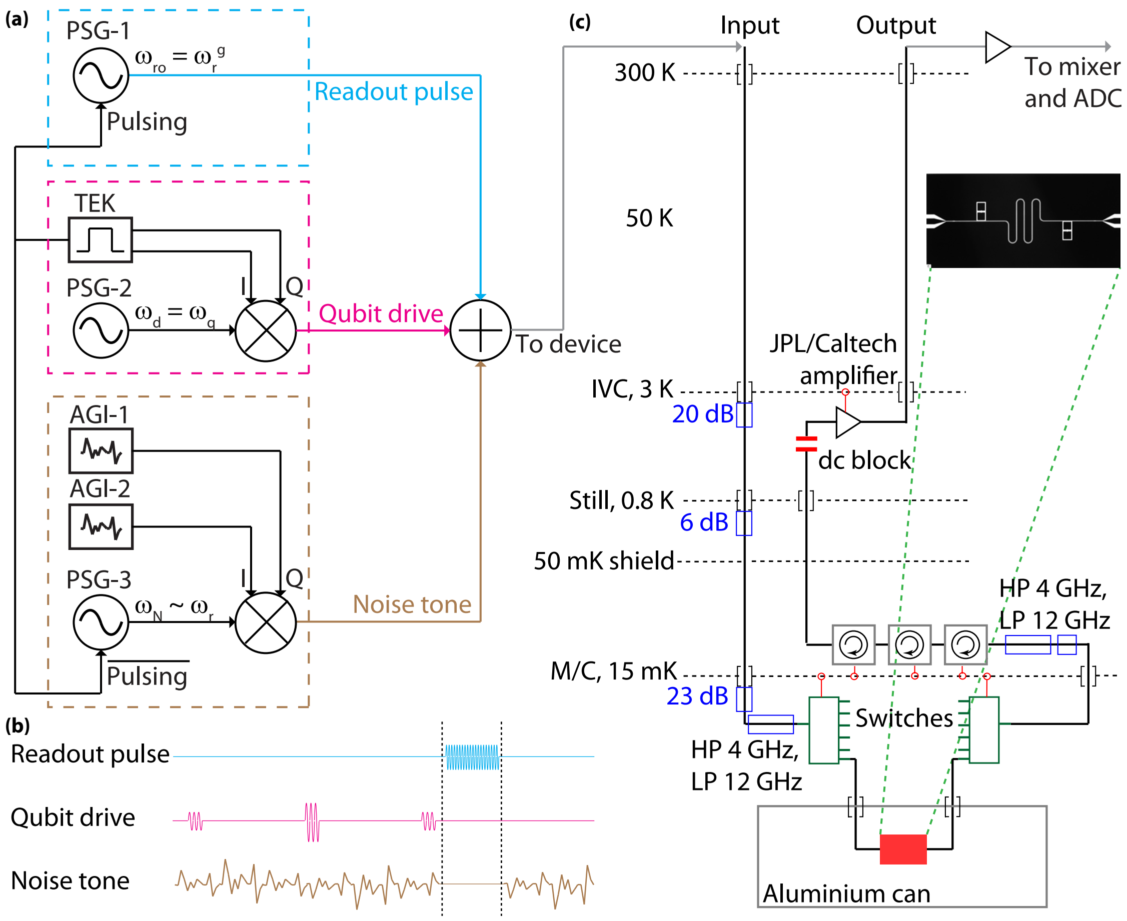

Supplementary Figure 2: **Experimental measurement schematic. a.** Diagram of the input-signal generation of readout (top panel, cyan), qubit drive (mid panel, magenta) and noise (bottom panel, brown) at room temperature. The signals are combined before being sent to the fridge. **b.** Relative timing of the signals generated in **a**. The dashed lines indicate the window within which the readout pulse is turned on while the noise is turned off. The representative qubit drive is a spin-echo pulse sequence, two $\pi/2$-pulses and a midpoint π-pulse. The actual pulses assume a Gaussian envelope. **c.** Wiring inside the refrigerator.

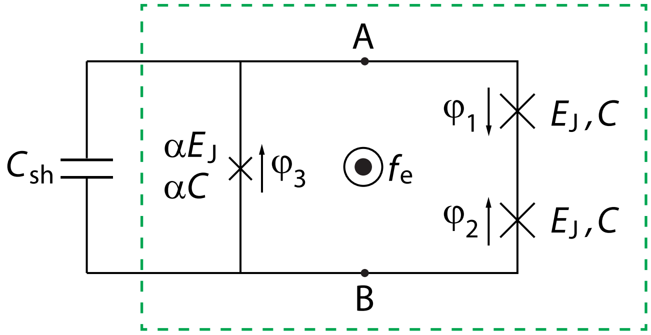

Supplementary Figure 3: **Circuit diagram of the C-shunt flux qubit.** Josephson junction 3 is α times the size of junctions 1 and 2. The external flux f_e is defined as the magnetic flux threading the loop formed by the three junctions. Nodes A and B represent the superconducting islands. The shunt capacitor has the dominant capacitance in the circuit. Node A is also capacitively coupled to a superconducting resonator (not drawn). The green dashed box highlights the part of the circuit resembling the conventional persistent-current flux qubit.

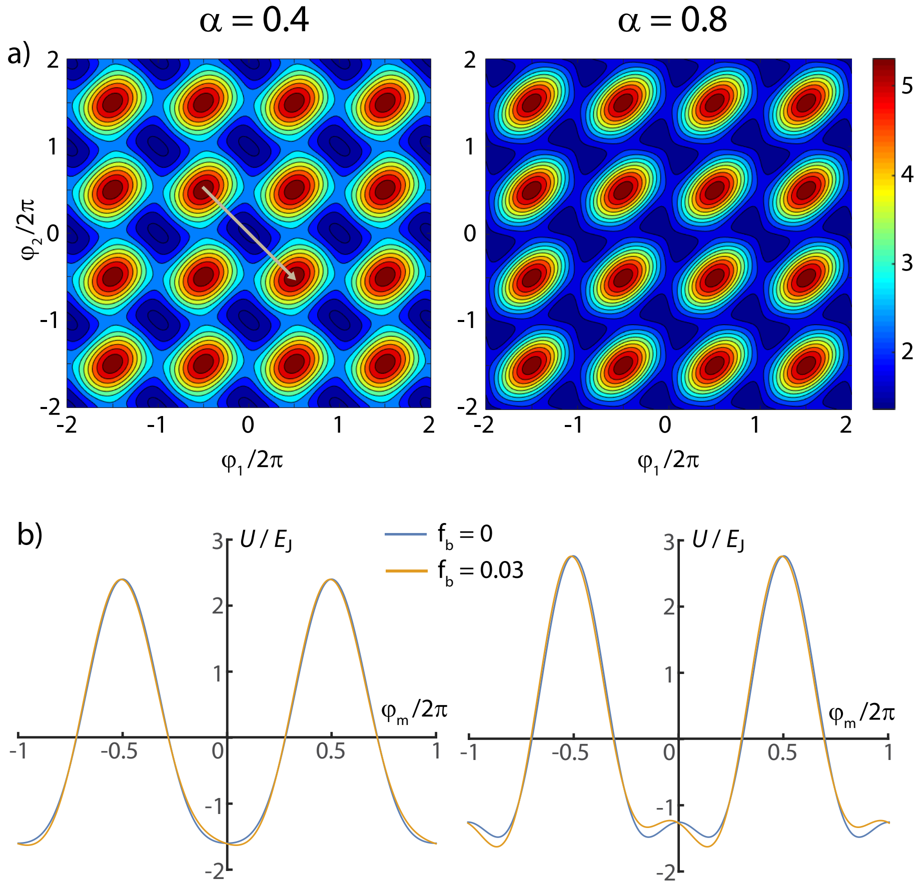

Supplementary Figure 4: **Potential profile of the C-shunt flux qubit. a.** A contour plot of the potential energy $U(\varphi_1, \varphi_2)$ at $f_\mathrm{b} = 0$ for $\alpha = 0.4$ (C-shunt flux qubit, on the left) and $\alpha = 0.8$ (traditional flux qubit, on the right). In the C-shunt case, the square-shaped area centered around $(\varphi_1, \varphi_2) = (k_1 \cdot 2\pi, k_2 \cdot 2\pi)$ marks the single well. In the traditional case, the figure-eight-shaped area marks the double well. The gray arrow indicates the $\varphi_\mathrm{m} = \varphi_1 - \varphi_2$ direction. **b.** The potential function along the φ_m direction at $\varphi_1 + \varphi_2 = 0$ at $f_\mathrm{b} = 0$ and $f_\mathrm{b} = 0.03$ for both $\alpha = 0.4$ and $\alpha = 0.8$ cases.

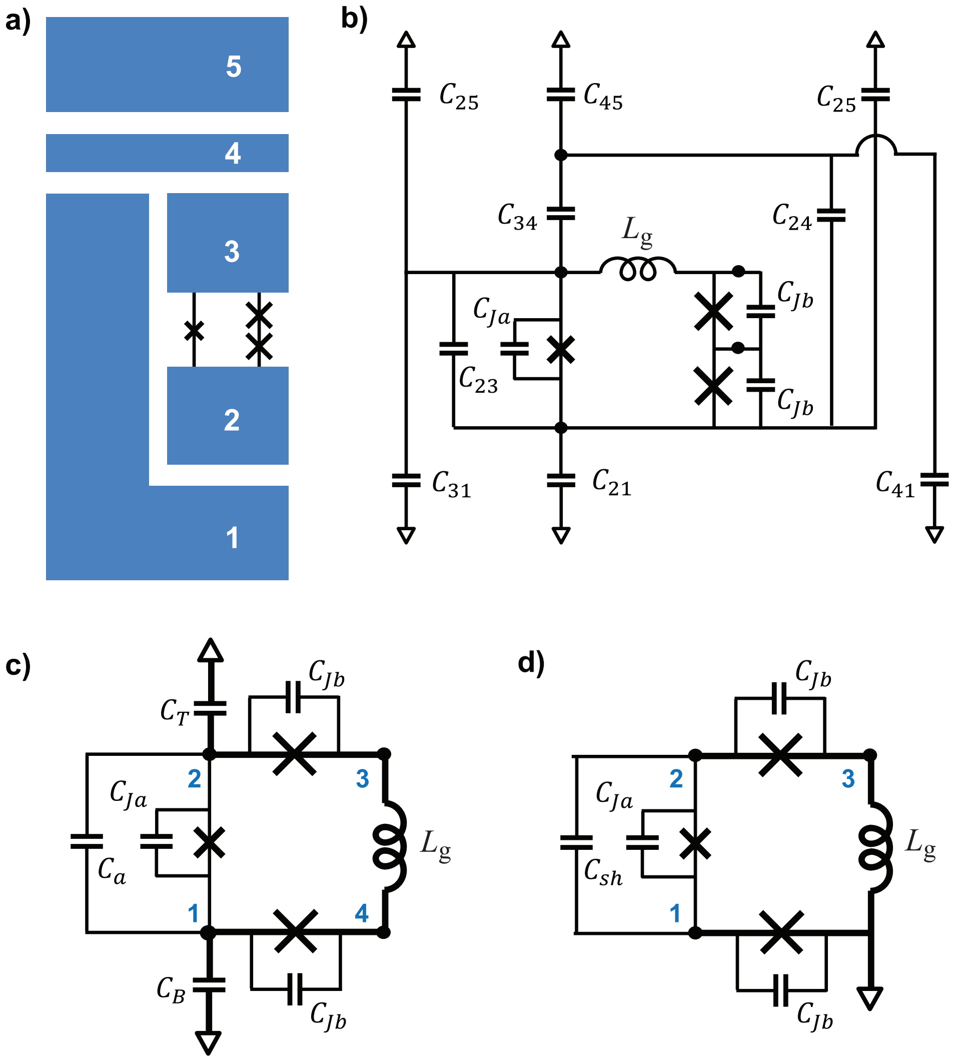

Supplementary Figure 5: **Circuit schematic for the C-shunt flux qubit and its reduction for use in simulation. a.** Schematic of the qubit-resonator system and the islands / nodes used in the simulation. For clarity the labeling follows that used for cQED transmons in Ref. 1. **b.** Equivalent circuit schematic with islands 1 and 5 grounded. Filled black circles indicate the five nodes that will be used in the full simulation. **c.** First reduction of the circuit in (b) containing four nodes (labeled in blue). See text for details. **d.** Second reduction of the circuit in (b) containing three nodes (labeled in blue). An effective shunt capacitance $C_{\rm sh}$ accounts for the capacitances $C_{\rm T}$, $C_{\rm B}$ and $C_{\rm a}$. See text for details.

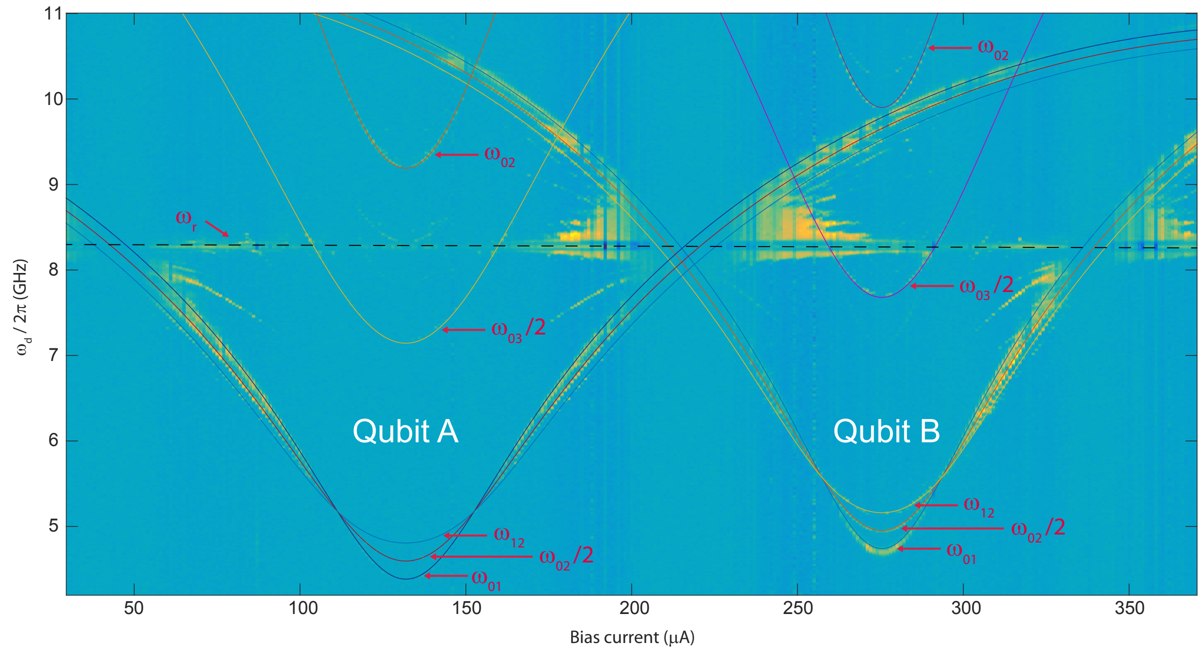

Supplementary Figure 6: **High-power spectra of qubits A and B with simulations to match the higher-level transitions.** The horizontal axis corresponds to the current in the "bobbin" coil of wire local to the qubits to apply a magnetic flux bias. Solid lines are simulation results that give the best match to the visible 0–1, 0–2 (two photon), 1–2, 0–3 (two photon) and 0–2 transitions. A precise matching to the experimental spectra required allowing these parameters to vary somewhat from their design values (Supplementary Table 1). Nonetheless, the simulation parameters (*e.g.,* J_c, $C_{\rm sh}$, *etc.*) are generally same for the two qubits, with the primary exception being the junction sizes.

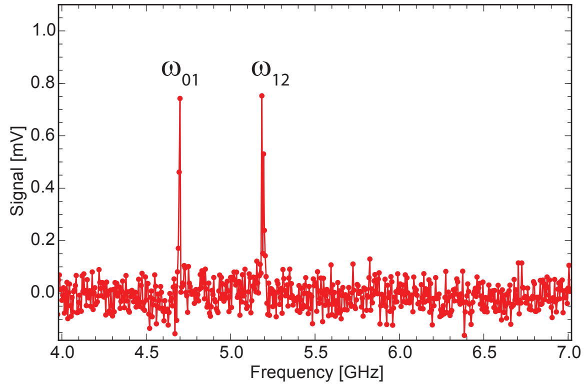

Supplementary Figure 7: **Two-tone spectroscopy of qubit B measured at the optimal bias point ($\Phi_e = \Phi_0/2$).** The data are taken by first applying a 20 ns microwave pulse at $\omega/2\pi = \omega_{01}/2\pi = 4.701$ GHz to drive the qubit to the $|1\rangle$ state, followed by a long (50 μs) low-power microwave tone. The frequency of the second tone is swept to perform spectroscopy. The resulting spectrum shows two peaks, one at 4.701 GHz that corresponds to driving the 1–0 transition, and one at 5.191 GHz that corresponds to the 1–2 transition. The measured anharmonicity is $(\omega_{12} - \omega_{01})/2\pi = 490$ MHz.

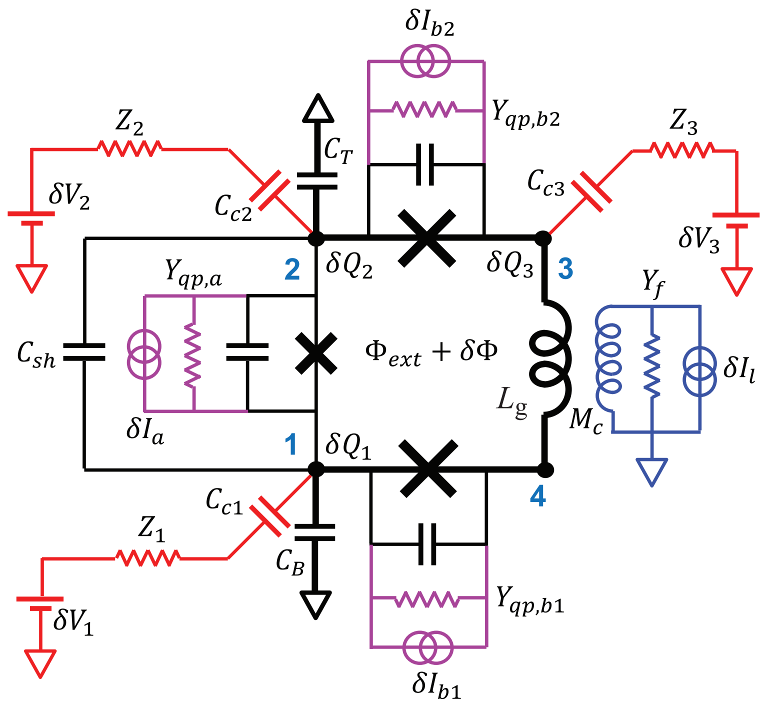

Supplementary Figure 8: **Circuit schematic used to account for electric and magnetic noise coupled to the qubit.** See main text Supplementary Note Supplementary Note 8 for details.

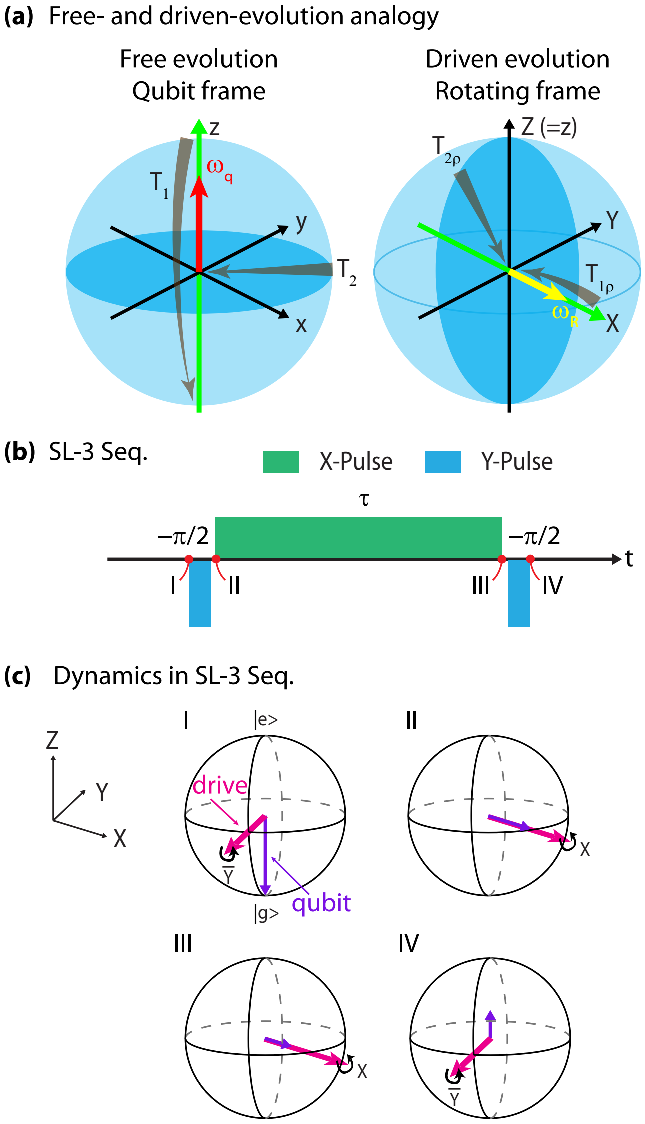

Supplementary Figure 9: **Spin locking and its implementation for noise spectroscopy. a.** Analogy between free- (left) and driven-evolution (right) dynamics. The free evolution is described in the qubit frame $\{\mathrm{x,y,z}\}$ while the driven evolution is described in the rotating frame $\{\mathrm{X,Y,Z}\}$. The two cases differ in the orientation and size of the static field, *i.e.,* "$\omega_{\mathrm{q}}\sigma_{\mathrm{z}}/2$" (red arrow) versus "$\omega_{\mathrm{R}}\sigma_{\mathrm{X}}/2$" (yellow arrow). The corresponding quantization axes (green arrows) and longitudinal/transverse relaxations (gray arrows) are defined with respect to the static field. **b.** Standard three-pulse spin-locking sequence (SL-3). The long driving pulse is 90°-phase-shifted from the $\pi/2$-pulses, and its length τ is the scanned parameter to record the rotating-frame relaxation. **c.** Bloch sphere representation of the rotating-frame qubit dynamics under SL-3. The purple arrows represent the polarization of the qubit states, while the magenta arrows indicate the driving-field orientation. The qubit is initially prepared in its ground state (I). The first $\pi/2$-pulse rotates the qubit by 90° into the equatorial plane (II). The second 90°-phase-shifted continuous driving pulse, of duration τ, is then aligned with the qubit state, effectively locking the qubit along X. During the pulse, the qubit undergoes relaxation in this rotating frame towards its steady state (III). The final $\pi/2$-pulse projects the remaining polarization onto Z (=z) for readout (IV).

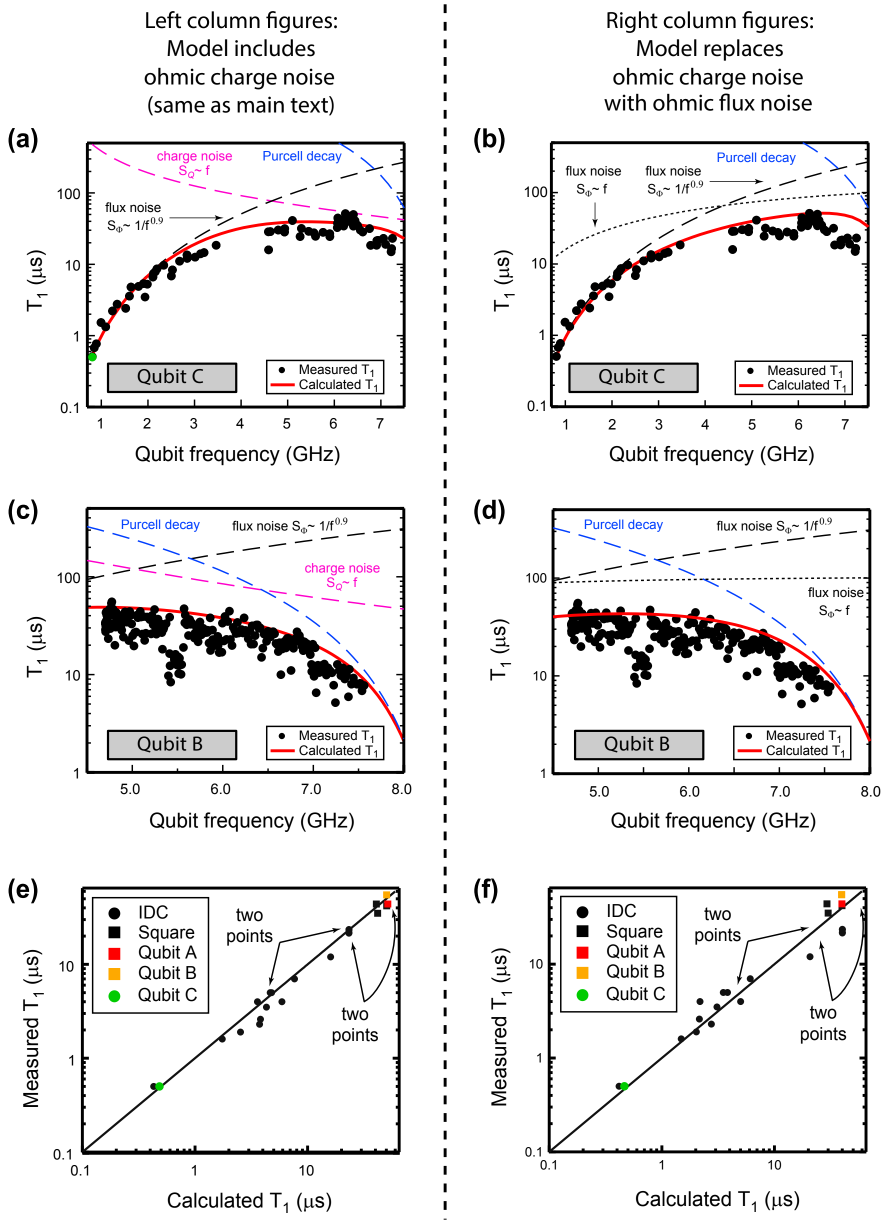

Supplementary Figure 10: **Comparison of a model using ohmic charge noise with one using ohmic flux noise.** Panels **a**, **c**, and **e** reproduce the panels in Fig. 3 from the main text, and the simulation traces contained therein are derived from a model involving ohmic charge noise, $1/f$ flux noise and the Purcell effect. For comparison, panels **b**, **d**, and **f** replace the ohmic charge noise with ohmic flux noise. The agreement with the data is similar between the two models, making it challenging to distinguish between ohmic charge noise and ohmic flux noise. It is only in panels **a** and **b** (device C) at frequencies above 6 GHz where the qubit is especially sensitive to ohmic charge noise that a plausible distinction can be observed.

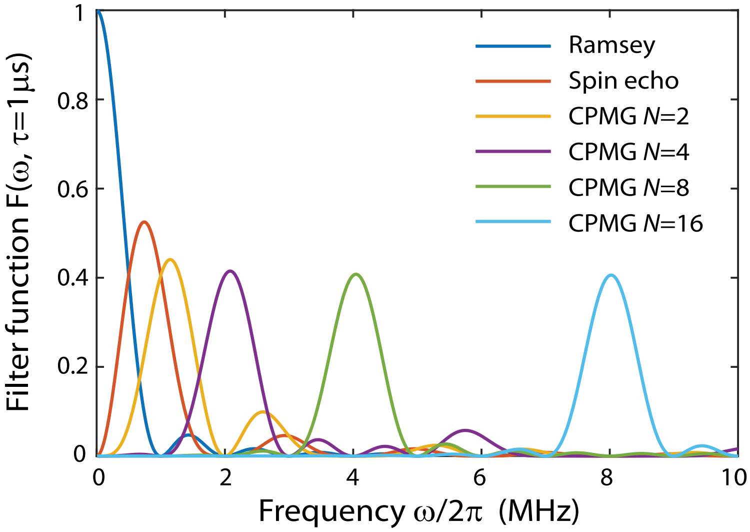

Supplementary Figure 11: **System filter functions corresponding to representative control sequences.** Filter functions of Ramsey, spin-echo and CPMG ($N = 2, 4, 8, 16$) sequences, assuming $\tau = 1\,\mu$s in all cases.

Sample		Shunt-cap. and Coupling				Junction Parameters						Qubit Parameters at $\Phi_\mathrm{b}=0$						Measured	
#	main text	cap type	gap (μm)	C_sh (fF)	β_c	α	J_c (μA/μm^2)	$E_{\mathrm{J}\alpha}$ (GHz)	$E_{\mathrm{C}\alpha}$ (GHz)	Δ (GHz)	$\mathcal{A}$ (GHz)	V_eg (μV)	I_p (nA)	T_1^Φ (μs)	T_1^Q (μs)	T_1^P (μs)	T_1^sim (μs)	T_1^meas (μs)	Δ (GHz)
1		IDC	40	21.3	0.19	0.55	6.7	139	0.74	3.36	6.15	4.28	194	4.4	21	742	3.6	4.0	4.4
2		IDC	40	21.3	0.19	0.55	6.7	139	0.74	3.36	6.15	4.28	194	4.8	19	742	3.8	2.6	4.9
3		IDC	20	21.3	0.19	0.59	6.6	127	0.75	1.50	8.37	2.46	206	1.8	155	3014	1.8	1.6	1.8
4		IDC	20	17.5	0.19	0.63	6.6	137	0.88	0.88	11.65	1.66	275	0.5	821	2894	0.5	0.5	0.7
5	C	IDC	40	9.0	0.31	0.68	6.6	133	1.46	0.82	17.8	1.76	275	0.5	685	2894	0.5	0.5	0.8
6		IDC	40	21.3	0.19	0.55	6.6	137	0.74	3.36	6.10	4.30	191	2.7	37	742	2.5	1.9	2.5
7		IDC	40	22.7	0.31	0.53	5.9	93.1	0.73	3.88	4.40	4.74	126	8.4	22	241	5.9	4.0	3.5
8		IDC	20	27.6	0.21	0.52	5.9	87.3	0.62	3.94	3.51	4.45	113	15	16	408	7.6	7.0	5.4
9		IDC	20	17.9	0.23	0.56	5.9	105	0.88	3.31	6.29	4.61	152	6.3	21	536	4.8	5.0	3.9
10		IDC	20	17.9	0.23	0.55	5.9	105	0.88	4.19	5.53	5.34	145	6.7	16	305	4.7	5.0	3.7
11		IDC	40	21.3	0.19	0.55	5.9	122	0.74	3.35	5.75	4.30	171	4.4	28	762	3.7	2.3	3.3
12		IDC	40	21.3	0.19	0.54	5.9	122	0.74	4.14	5.05	4.90	163	5.8	18	467	4.3	3.5	4.1
13		IDC	20	26.8	0.15	0.52	3.0	44.3	0.64	3.19	2.73	4.05	61.8	32	33	1378	16	12	3.2
14		IDC-R	10	51.4	0.14	0.43	2.4	36.2	0.35	3.94	0.88	3.43	43.9	82	34	643	23	23	4.2
15		IDC-R	10	51.4	0.14	0.43	2.4	36.2	0.35	3.94	0.88	3.43	43.9	82	34	643	23	22	4.2
16		IDC-R	30	51.9	0.14	0.43	2.4	36.2	0.35	3.94	0.87	3.43	43.9	82	34	643	23	24	4.2
17		IDC-R	30	51.9	0.14	0.43	2.4	36.2	0.35	3.94	0.87	3.43	43.9	82	34	643	23	22	4.2
18		SQR	40	51.0	0.089	0.43	2.9	43.0	0.35	4.33	0.88	3.60	50.3	68	147	408	42	35	4.7
19		SQR	40	51.0	0.089	0.43	2.9	43.0	0.35	4.68	0.88	3.74	50.1	75	124	330	41	44	5.1
20		SQR	40	51.0	0.089	0.42	2.4	36.2	0.35	3.96	0.80	3.45	43.9	79	184	567	50	42	4.0
21	A	SQR	40	51.0	0.089	0.43	2.4	36.2	0.35	3.96	0.91	3.45	43.9	85	169	567	52	44	4.4
22	B	SQR	40	51.0	0.089	0.42	2.4	36.2	0.35	4.29	0.83	3.60	43.5	92	145	474	50	55	4.7

Supplementary Table 1: Parameters for 22 qubits studied in this work, spanning shunt capacitance values $C_\mathrm{sh} = 9 \ldots 52$ fF. Color-highlighting corresponds to the samples A, B and C presented in detail in the main text. All parameters are design values or are derived from simualtion, except for the critical current density J_c and the measured relaxation time T_1^meas. Three capacitor types were studied: IDC is "interdigital capacitor"; IDC-R is an IDC with slightly rounded corners (negligible when compared with actualized standard IDCs, but included here for completeness); and SQR is square capacitor shape. Gap is the spacing between capacitor features (*e.g.,* IDC fingers). β_c quantifies the coupling strength between the qubit and the resonator. $E_{\mathrm{J}\alpha}$ and $E_{\mathrm{C}\alpha}$ are respectively the Josephson energy and charging energy of the small junction. Δ is the qubit frequency at the qubit flux-insensitive (degeneracy) point $\Phi_\mathrm{b} = 0$. $\mathcal{A}$ is the anharmonicity. V_eg is the simulated island node voltage (related to the charge transverse matrix element). I_p is the simulated persistent current (related to the flux transverse matrix element). T_1^Φ, T_1^Q, and T_1^P are respectively the simulated T_1 values due to flux noise, charge noise, and the Purcell effect (all at $\Phi_\mathrm{b} = 0$). T_1^sim is the net simulated T_1 value due to these processes, and and T_1^meas is the measured relaxation time at $\Phi_\mathrm{b} = 0$.

Supplementary Note 1 Materials and Fabrication of the High-Q Capacitor

The capacitively-shunted flux qubits studied in this work were prepared using the following steps:

1. Growth and patterning of high-quality-factor (high-Q) aluminum films using molecular beam epitaxy (MBE).
2. Patterning and evaporation of the superconducting qubit loop and Josephson junctions.
3. Dicing and packaging.

Supplementary Note 1.1 Growth and patterning of high-Q aluminum

High-Q aluminum films were deposited on 50-mm C-plane sapphire wafers in a Veeco GEN200 MBE system with a growth chamber base pressure of 10^{-11} torr. The wafers were cleaned in piranha solution (sulfuric acid and hydrogen peroxide) prior to loading into the MBE system. The wafers were annealed in the MBE system at $900\,^{\circ}$C to facilitate outgassing and sapphire surface reconstruction, after which 250 nm of aluminum was deposited at a growth rate of 0.025 nm/s and a substrate temperature of $150\,^{\circ}$C (Supplementary Figure 1a).

The high-Q aluminum was patterned using contact lithography and wet-etched using Aluminum Etchant - Type A (Transene Company, Inc.) into the following device features (Supplementary Figure 1b): shunt capacitors, coplanar waveguide (CPW) resonators, ground planes, and optical alignment marks.

Supplementary Note 1.2 Patterning the qubit loop and Josephson junctions

The qubit loop and junctions were formed using double-angle evaporation of aluminum through Dolan-style bridges (Supplementary Figure 1c) [2]. The free-standing bridges were realized using a bilayer mask comprising a germanium hard mask on top of a sacrificial MMA/MAA layer [MicroChem methyl methacrylate (MMA (8.5)/MAA EL9)]. The qubit loop and junctions were patterned using electron-beam lithography (Vistec EBPG5200) and ZEP520A resist (ZEONREX Electronic Chemicals). This pattern was transferred into the Ge layer using a CF_4 plasma, and the underlying MMA/MAA resist was under-etched using an oxygen plasma to create free-standing bridges. Prior to the aluminum evaporation, an *in situ* argon ion milling was used to clean exposed contact points on the MBE aluminum to ensure superconducting contact with the evaporated aluminum. The qubit loops and junctions were realized with two separate angle-evaporated aluminum layers; between the two aluminum evaporation steps, static oxidation conditions were used to prepare junctions with a certain critical current density.

Supplementary Note 1.3 Dicing and packaging

Devices were diced into 2.5 x 5 mm^2 chips (as shown in the manuscript in Fig. 1a) that were mounted into gold-plated copper packages. Aluminum wirebonds were used for both signal and ground connections between the device and package, as well as to connect the ground planes of the CPW resonator to prevent slotline modes.

Supplementary Note 2 Measurement Set-up and Protocol

We performed our experiments at MIT in a Leiden Cryogenics (CF-450) dilution refrigerator with a base temperature of 15 mK. The device was magnetically shielded with a superconducting can surrounded by a Cryoperm-10 cylinder. All electrical leads were attenuated and/or filtered to reduce noise.

Supplementary Note 2.1 Outside the dilution refrigerator

The electronic setup for generating the readout-pulses, control-pulses, and artificial photon noise is shown in Supplementary Figure 2a. All time-sensitive instruments are synchronized with a Stanford Research Systems FS725 Rubidium Frequency Standard. The readout-pulse is generated by an Agilent 8267D Vector Signal Generator (PSG), gated by a Tektronix 5014b arbitrary waveform generator (TEK). This pulse is typically a few microseconds long, and the tone is set at the resonator frequency dressed by the qubit in its ground state ($\omega_{\rm r}^{\rm g}/2\pi$). The control-pulse envelope for driving the qubit is generated by the TEK and mixed with a qubit-frequency tone ($\omega_{\rm q}/2\pi$) from a second PSG using its internal I-Q mixer. These pulses are further gated to reduce unwanted mixer leakage. Artificial photon noise is generated by up-converting (quadrature-mixing) 80-megahertz-wide white-noise signals from two Agilent 33250A arbitrary waveform generators (AGI) with a tone near the cavity frequency from a third PSG. We confirmed that the noise source behaved consistently using either one port or both ports of the I-Q mixer. For example, with equal powers applied to each port, the output is simply a doubling of the photon noise power generated from a single port. The carrier frequency for the noise is chosen to be $\sim$10 MHz away from the readout frequency, much larger than the cavity linewidth. In addition, we gate the noise off during the readout pulse using the TEK. The three signals are combined (relative timing illustrated in Supplementary Figure 2b) and sent to the input port on the refrigerator. This sequence constitutes a single experimental trial, and it is repeated typically 10,000 times with a period of 200-400 μs.

Supplementary Note 2.2 Inside the dilution refrigerator

The device is enclosed in a copper package, which is itself mounted inside an aluminum box to shield the device from external electromagnetic radiation and magnetic field fluctuations (Supplementary Figure 2c). A small coil antenna is mounted under the package lid and is used to bias the qubit with a static magnetic field (not shown). A Yokogawa 7651 dc source provides the bias current to the coil using twisted pair wires passing through an RC low-pass filter with cutoff around 100 kHz. On the input side, there is a total attenuation of 49 dB arising from discrete attenuators (XMA Corporation) at various temperature stages. In addition, there is a relatively small amount of distributed attenuation due to loss in the coaxial cables. On both the input and output sides, a high-pass filter (RLC F-18948, 4 GHz cutoff) and a low-pass filter (RLC L-3615, 12.4 GHz cutoff) provide a net 4-12.4 GHz passband. After the output filters, 3 isolators (Quinstar / Pamtech, model CWJ1019KS414, 3-12 GHz, with approximately 15-20 dB isolation each) are mounted on the mixing chamber. The output signal is amplified by a JPL/Caltech cryogenic preamplifier (1-12 GHz, 30 dB gain). The output port outside the refrigerator is followed by a room-temperature amplifier (MITEQ, AMF-5D-00101200-23-10P, 0.1-12 GHz, 43 dB gain) before mixing with a local oscillator (LO) tone ($\omega_{\rm ro}/2\pi - \omega_{\rm LO}/2\pi = 50\,{\rm MHz}$) for heterodyne detection (not shown). After the mixers, the signal is digitized using an Acquiris U1084A analog-to-digital converter (ADC) and digitally demodulated to extract the amplitude and phase of the readout signal for qubit-state estimation.

Supplementary Note 3 Two-Level-System Model of C-Shunt Flux Qubit

Supplementary Note 3.1 Parameterization of the two-level-system Hamiltonian

In the main text, we elect to parameterize the C-shunt flux qubit using the familiar notation of the conventional flux qubit [3], albeit with important generalizations due to the influence of higher energy levels. In the laboratory-frame, the two-level-system Hamiltonian for an individual C-shunt flux qubit near flux-degeneracy and coupled to a CPW resonator is:

$$\hat{\mathcal{H}} = \frac{\hbar}{2}\left[\Delta\left(\Phi_{\mathrm{b}}\right)\ \hat{\sigma}_{\mathrm{x}} + \varepsilon\left(\Phi_{\mathrm{b}}\right)\ \hat{\sigma}_{\mathrm{z}}\right] + \hbar\omega_{\mathrm{r}}(\hat{a}^{\dagger}\hat{a} + 1/2) + \hbar g\left(\Phi_{\mathrm{b}}\right)\hat{\sigma}_{\mathrm{y}}(\hat{a}^{\dagger} + \hat{a}). \tag{1}$$

Here, the three terms are respectively the qubit, resonator, and qubit-resonator interaction Hamiltonians, $\hat{\sigma}_{\mathrm{x,y,z}}$ are the Pauli operators for the qubit, $\Phi_{\mathrm{b}} \equiv \Phi_{\mathrm{e}} - \Phi_0/2$ is the flux bias due to an external magnetic flux Φ_{e} ($\Phi_0 \equiv h/2e$ is the superconducting flux quantum), $\hat{a}^{\dagger}(\hat{a})$ is the raising(lowering) operator for resonator photons, ω_{r} is the resonator angular frequency and g is the qubit-resonator coupling angular frequency. In this coordinate system, the qubit shunt-capacitor couples transversally to the qubit through $\hat{\sigma}_{\mathrm{y}}$. Similarly, charge fluctuations connect through $\hat{\sigma}_{\mathrm{y}}$.

Within our parametrization, the circulating current states of the C-shunt flux qubit have energies $\pm\hbar\,\varepsilon(\Phi_{\mathrm{b}})/2 \equiv \pm I_{\mathrm{m}}(\Phi_{\mathrm{b}})\,\Phi_{\mathrm{b}}$, where $I_{\mathrm{m}}\left(\Phi_{\mathrm{b}}\right) \equiv \left[I_{\mathrm{p1}}\left(\Phi_{\mathrm{b}}\right) - I_{\mathrm{p0}}\left(\Phi_{\mathrm{b}}\right)\right]/2$ is related to the difference in the flux-dependent circulating (persistent) currents $I_{\mathrm{p0}}(\Phi_{\mathrm{b}})$ and $I_{\mathrm{p1}}(\Phi_{\mathrm{b}})$, and these states hybridize with a flux-dependent energy $\hbar\Delta(\Phi_{\mathrm{b}})$. With this generalization, a flux-dependent offset energy $\hbar\varepsilon_{\mathrm{off}}\left(\Phi_{\mathrm{b}}\right) \equiv I_{\mathrm{off}}\left(\Phi_{\mathrm{b}}\right)\Phi_{\mathrm{b}}$ is removed, where $I_{\mathrm{off}}\left(\Phi_{\mathrm{b}}\right) \equiv \left[I_{\mathrm{p1}}\left(\Phi_{\mathrm{b}}\right) + I_{\mathrm{p0}}\left(\Phi_{\mathrm{b}}\right)\right]/2$. In addition to their flux dependence, the currents $I_{\mathrm{p0,p1}}(\Phi_{\mathrm{b}})$ generally do not have the same magnitude, nor need they have opposing sign. This is in marked contrast to the conventional flux qubit, where $I_{\mathrm{p0}} = -I_{\mathrm{p1}} \equiv I_{\mathrm{p}}$ with both I_{p} and Δ being independent of flux within a certain region about $\Phi_{\mathrm{b}} = 0$. The fact that $I_{\mathrm{p0,p1}}\left(\Phi_{\mathrm{b}}\right)$ and $\Delta\left(\Phi_{\mathrm{b}}\right)$ are flux-dependent for the C-shunt flux qubit reflects the non-negligible role of its higher energy levels. Consequently, we numerically diagonalize the full qubit-resonator Hamiltonian for parameter extraction and modeling.

The extent to which higher levels of the qubit influence the parameters in Eq. (1) is determined by the ratio $\omega_{\mathrm{p}}^{(k)}/\omega_{\mathrm{q}}$ where $\omega_{\mathrm{q}} = \sqrt{\varepsilon(\Phi_{\mathrm{b}})^2 + \Delta(\Phi_{\mathrm{b}})^2}$ is the qubit frequency, and $\omega_{\mathrm{p}}^{(k)}$ are the plasma frequencies of the additional oscillator-like modes of the circuit. For conventional flux qubits, these modes are typically much higher in energy than the qubit excited state and can be neglected. However, as the shunt capacitance is increased, at least one of these modes shifts to lower frequency and can eventually become comparable to the qubit frequency itself. For example, one of these modes is the small junction "plasma frequency" $\omega_{\mathrm{p},\alpha} = 1/\sqrt{L_{\mathrm{J},\alpha}C_{\alpha}}$ determined by its Josephson inductance $L_{\mathrm{J},\alpha}$ and its total capacitance C_{α} (including the shunt capacitor). This is the frequency of the oscillator-like degree of freedom for the qubit in each of the two wells of its double-well potential profile (each well is associated with a circulating persistent current $I_{\mathrm{p0,p1}}$), and this frequency is ideally far above ω_{q}. For a conventional flux qubit, $\omega_{\mathrm{p}}^{(k)}/\omega_{\mathrm{q}} \gtrsim 10$, and the parameters in Eq. (1) are largely flux-independent over a wide range of flux bias about $\Phi_0/2$ (see Ref. 4 for experiments that study the flux range required to access higher levels for a particular conventional flux qubit). As the ratio $\omega_{\mathrm{p}}^{(k)}/\omega_{\mathrm{q}}$ is decreased, the excited states of the plasma mode shift closer to the two qubit levels and quantum mixing (hybridization) occurs, resulting in increased quantum fluctuations of the current. Correspondingly, there is a decrease in the range in flux over which the parameters in Eq. (1) are essentially flux independent. Eventually, when $\omega_{\mathrm{p}}^{(k)}/\omega_{\mathrm{q}} \gtrsim 1$, the qubit can be descried as a weakly-harmonic oscillator (much like a transmon), where the only remnants of the persistent currents are small, state-dependent displacements of the oscillator current from zero, which are much smaller than their quantum fluctuations. Typically, the resulting weak anharmonicity is inverted relative to that of the transmon. The highest-coherence C-shunt flux qubits considered in this work are in an intermediate regime, having $\omega_{\mathrm{p}}^{(k)}/\omega_{\mathrm{q}} \gtrsim 2$. Their anharmonicity is around 500-900 MHz for the longest lived devices, generally larger than the 200-300 MHz observed in transmons.

The role of higher energy levels is described in Section Supplementary Note 4, along with a comparison between the conventional and C-shunt flux qubits. Ultimately, to account for the higher energy levels, the two-

level parametrization must be deduced from experiment, an analytic treatment (Section Supplementary Note 4), or simulations (Section Supplementary Note 5).

Supplementary Note 3.2 Dispersive two-level-system Hamiltonian

The unitary $\hat{U}_1 = \exp\left[\mathrm{i}\,(\frac{\pi}{2}-\theta)\hat{\sigma}_\mathrm{y}/2\right]$ rotates Eq. (1) from the laboratory frame to the qubit frame,

$$\hat{U}_1\hat{\mathcal{H}}\hat{U}_1^\dagger = \hbar\omega_\mathrm{q}\hat{\sigma}_\mathrm{z}/2 + \hbar\omega_\mathrm{r}(\hat{a}^\dagger\hat{a}+1/2) + \hbar g\hat{\sigma}_\mathrm{y}(\hat{a}^\dagger+\hat{a})\ , \tag{2}$$

where $\theta = \arctan(\varepsilon/\Delta)$. The transverse qubit-resonator coupling is unaffected by the transformation, since the flux bias Φ_b rotates the qubit quantization axis within the x–z plane (laboratory frame) and the capacitive coupling is transverse to this plane.

In the dispersive regime, $|\delta_{\mathrm{q,r}}| = |\omega_\mathrm{q}-\omega_\mathrm{r}| \gg g$, after a second unitary transformation $\hat{U}_2 = \exp\left[-\mathrm{i}\,\frac{g}{\Delta_{\mathrm{q,r}}}(\hat{a}\hat{\sigma}_+ + \hat{a}^\dagger\hat{\sigma}_-)\right]$, the Hamiltonian can be approximated:

$$\hat{\mathcal{H}}_\mathrm{disp} = \hat{U}_2\hat{U}_1\hat{\mathcal{H}}\hat{U}_1^\dagger\hat{U}_2^\dagger \approx \hbar\omega_\mathrm{q}\hat{\sigma}_\mathrm{z}/2 + \hbar\omega_\mathrm{r}(\hat{a}^\dagger\hat{a}+1/2) + \hbar\,2\chi(\hat{a}^\dagger\hat{a}+1/2)\hat{\sigma}_\mathrm{z}/2\ . \tag{3}$$

Here, $\omega_\mathrm{q} = \sqrt{\varepsilon(\Phi_\mathrm{b})^2+\Delta(\Phi_\mathrm{b})^2}$ is the qubit angular frequency and $\chi(\Phi_\mathrm{b})$ is related to the qubit-state-dependent dispersive shift $2\chi(\Phi_\mathrm{b}) = g^2(\Phi_\mathrm{b})/\delta_{\mathrm{q,r}}(\Phi_\mathrm{b})$ of the resonator used for readout. The last term includes the Stark shift $\Delta_\mathrm{Stark} = 2\chi(\Phi_\mathrm{b})\,\hat{n}$ due to the resonator photon number $\hat{n} = \hat{a}^\dagger\hat{a}$ and the Lamb shift $\Delta_\mathrm{Lamb} = \chi(\Phi_\mathrm{b})$ due to the resonator zero-point energy. Higher levels of the qubit generally play an important role in constructing the flux-dependent dispersive shift. While it is straightforward to measure this experimentally, one must go beyond a two-level approximation to calculate it accurately.

Supplementary Note 4 Analytic Treatment and Comparison of the Conventional and C-Shunt Flux Qubits

Supplementary Note 4.1 Conventional persistent-current flux qubit

Before developing the system model for the C-shunt flux qubit, we review the conventional persistent-current flux qubit as presented in Ref. [3]. The conventional flux qubit circuit is illustrated in Supplementary Figure 3 (dashed box). The qubit loop is interrupted by three Josephson junctions, and φ_i ($i = 1, 2, 3$) are the associated gauge-invariant phase differences. Two of the junctions have the same critical current I_c and junction capacitance C, and thus the same Josephson energy $E_\mathrm{J} \equiv I_\mathrm{c}\Phi_0/2\pi$ and charging energy $E_\mathrm{C} \equiv e^2/2C$. The third junction is smaller in area by a factor α, resulting in a reduced critical current αI_c and reduced junction capacitance αC. The corresponding energy scales are αE_J and E_C/α.

The flux quantization condition gives $\varphi_1 - \varphi_2 + \varphi_3 = 2\pi f_\mathrm{e}$, where $f_\mathrm{e} \equiv \Phi_\mathrm{e}/\Phi_0$ is the external magnetic flux threading the loop in units of the superconducting flux quantum $\Phi_0 = h/2e$. When $\alpha > 0.5$ and $f_\mathrm{e} \approx 0.5$, the potential energy of the qubit assumes a double-well profile. The wells are associated with clockwise and counterclockwise circulating currents tunable by the applied magnetic flux. These diabatic circulating-current states tunnel-couple with a strength depending on the height of the inter-well barrier or the scale factor α.

Tuning f_e tilts the double well potential. In the vicinity of $f_\mathrm{e} \approx 0.5$, the circulating currents are of opposite sign and essentially equal magnitude. Consequently, in this limit, higher energy levels play little role and the two-level approximation is a good approximation to the full Hamiltonian. However, even for the conventional flux qubit, higher energy levels become important for flux biases far from $f_\mathrm{e} \approx 0.5$, where the double-well potential is tilted to such a degree that its higher levels influence the circulating currents [4,5]. The region about $f_\mathrm{e} \approx 0.5$ for which the two-level system is a good approximation is reduced as α decreases.

Supplementary Note 4.2 C-shunt flux qubit: quasistatics

The C-shunt flux qubit has three features that are distinctly different from the conventional design: (i) lower I_c, (ii) lower α, typically $\alpha < 0.5$ (defined for three-junction qubits) and (iii) an additional large capacitor $C_{\rm sh} = \zeta C$ ($\zeta \gg 1$) shunting the smaller junction. As we show below, both (i) and (ii) lead to reduced sensitivity to flux noise, while (iii) reduces sensitivity to charge noise.

Following a recipe similar to that presented in Ref. [3], the three-junction capacitively-shunted flux qubit can be described by a Hamiltonian consisting of the kinetic and potential part:

$$\begin{aligned}
\hat{\mathcal{H}} &= \hat{T} + \hat{U} \ , \\
\hat{T} &= \frac{1}{2}(\hat{\mathbf{Q}} + \hat{\mathbf{q}})^{\rm T}\, \mathbf{C}^{-1} (\hat{\mathbf{Q}} + \hat{\mathbf{q}}) \ , \\
\hat{U} &= E_{\rm J}\{2 + \alpha - \cos\varphi_1 - \cos\varphi_2 - \alpha\cos(2\pi f_{\rm e} + \varphi_1 - \varphi_2)\} \ .
\end{aligned} \tag{4}$$

In the kinetic part, $\hat{\mathbf{Q}}$ and $\hat{\mathbf{q}}$ are the charges and induced charges on the islands:

$$\hat{\mathbf{Q}} = -{\rm i}\, 2e \begin{pmatrix} \frac{\partial}{\partial\varphi_1} \\ \frac{\partial}{\partial\varphi_2} \end{pmatrix} \ \text{and} \ \ \hat{\mathbf{q}} = \begin{pmatrix} q_{\rm A} \\ q_{\rm B} \end{pmatrix} \ ; \quad \mathbf{C} = C \begin{pmatrix} \zeta + 1 + \alpha & -(\zeta+\alpha) \\ -(\zeta+\alpha) & \zeta + 1 + \alpha \end{pmatrix} \ . \tag{5}$$

The kinetic energy represents the total electrostatic energy stored in the capacitors, and is dominated by the shunt capacitor, since $\zeta \gg 1, \alpha$. The shunt capacitor largely reduces the effective charging energy, causing the system less sensitive to charge fluctuations. Compared with the circuit model in Ref. [3], there is no explicit gate electrode in our circuit. The induced charge, however, is included for modelling the charge noise, and will be discussed later in this section. In the static case, $q_{\rm A} = q_{\rm B} = 0$. The potential energy sums up the Josephson energy stored in the junctions. It assumes a two-dimensional periodic profile. When $\alpha \leq 0.5$, as in this C-shunt design, there is only one well in each unit cell (see Supplementary Figures 4a and 4b), a distinction from the double-well profile in the conventional case. This leads to a smaller circulating current for the C-shunt flux qubit, reducing its sensitivity to flux fluctuations.

By choosing $\varphi_{\rm p} = (\varphi_1 + \varphi_2)/2$ and $\varphi_{\rm m} = (\varphi_1 - \varphi_2)/2$ as coordinates and using the Cooper-pair number operators $\hat{n}_\sigma = -{\rm i}\,\partial/\partial\varphi_\sigma$ ($\sigma = {\rm p, m}$), we have the reduced Hamiltonian

$$\hat{\mathcal{H}} = \frac{1}{2}E_{\rm C,p}\hat{n}_{\rm p}^2 + \frac{1}{2}E_{\rm C,m}\hat{n}_{\rm m}^2 + E_{\rm J}\{2 + \alpha - 2\cos\varphi_{\rm p}\cos\varphi_{\rm m} - \alpha\cos(2\pi f_{\rm e} + 2\varphi_{\rm m})\} \ , \tag{6}$$

where $E_{\rm C,p} = e^2/(C/2)$ and $E_{\rm C,m} = e^2/C(\zeta + \alpha + 1/2)$ are the effective charging energy for the p-mode and m-mode respectively. Ideally, because the introduction of the shunt capacitor ensures that $E_{\rm C,p} \approx 2\zeta E_{\rm C,m} \gg E_{\rm C,m}$, we can safely omit the p-mode, since the characteristic frequency $\Omega_{\rm p} = \sqrt{E_{\rm C,p}E_{\rm J,p}}/\hbar \approx 2\pi \times 50\,{\rm GHz}$ is much higher. Here, $E_{\rm J,p} = 2E_{\rm J}$ is the effective p-mode Josephson energy. In a realistic design, one must also assess the role of additional plasma modes, for example, due to capacitance to ground, and their characteristic frequencies. The simplified Hamiltonian becomes

$$\hat{\mathcal{H}}_{\rm m} = \frac{1}{2}E_{\rm C,m}\hat{n}_{\rm m}^2 + E_{\rm J}\{-2\cos\varphi_{\rm m} + \alpha\cos(2\pi f_{\rm b} + 2\varphi_{\rm m})\} \ , \tag{7}$$

where $f_{\rm b} = f_{\rm e} - 0.5$ is the reduced external flux bias away from one-half flux quantum.

To understand the energy structure of the system, we Taylor-expand the potential function $U(\varphi_{\rm m}) = E_{\rm J}\{-2\cos\varphi_{\rm m} + \alpha\cos(2\pi f_{\rm b} + 2\varphi_{\rm m})\}$ around the well minimum $\varphi_{\rm m}^*$,

$$U(\varphi_{\rm m}) = \sum_{k=0}^{\infty} U^{(k)}(\varphi_{\rm m} - \varphi_{\rm m}^*)^k \ , \tag{8}$$

where $U^{(k)} = (1/k!)(\partial^k U/\partial\varphi_{\rm m}^k|_{\varphi_{\rm m}=\varphi_{\rm m}^*})$. For a given $f_{\rm b}$, $\varphi_{\rm m}^*$ is single-valued within the range $\varphi_{\rm m} \in [-\pi, \pi]$ and obtainable by solving the equation $U'(\varphi_{\rm m}^*) = 0$. By definition, $U^{(0)}$ is a constant and $U^{(1)} = 0$. Therefore, we can rewrite Eq. (7) as:

$$\begin{aligned}\hat{\mathcal{H}}_{\rm m} &= \hat{\mathcal{H}}_0 + \hat{V} \\ &= \left(\frac{1}{2}E_{\rm C,m}\hat{n}_{\rm m'}^2 + \frac{1}{2}E_{\rm J,m}\hat{\varphi}_{\rm m'}^2\right) + \sum_{k=3}^{\infty} U^{(k)}\hat{\varphi}_{\rm m'}^k, \\ &= \hbar\Omega_{\rm m}^{(0)}(\hat{b}^\dagger\hat{b} + \frac{1}{2}) + \sum_{k=3}^{\infty} U^{(k)}\varphi_{\rm z}^k(\hat{b} + \hat{b}^\dagger)^k \ . \end{aligned} \tag{9}$$

On the second line, $E_{\rm J,m} = 2U^{(2)}$ is the effective Josephson energy, $\hat{\varphi}_{\rm m'} = \varphi_{\rm m'} = \varphi_{\rm m} - \varphi_{\rm m}^*$ and $\hat{n}_{\rm m'} = -{\rm i}\,\partial/\partial\varphi_{\rm m'}$. We group the quadratic potential term with the kinetic part so that their combination $\mathcal{H}_0$ describes a one-dimensional harmonic oscillator. The last line expresses the Hamiltonian in terms of the raising ($\hat{b}^\dagger$) and lowering ($\hat{b}$) operators of the harmonic oscillator. The oscillator frequency is $\Omega_{\rm m}^{(0)} = \sqrt{E_{\rm C,m}E_{\rm J,m}}/\hbar$. The quantum ground-state phase uncertainty is $\varphi_{\rm z} = (E_{\rm C,m}/4E_{\rm J,m})^{1/4}$ [6]. After the replacement $\hat{\varphi}_{\rm m'} = \varphi_{\rm z}(\hat{b} + \hat{b}^\dagger)$, the perturbation matrix V can be conveniently computed in the space spanned by the Fock states of $\mathcal{H}_0$. Note that $\hbar\Omega_{\rm m}^{(0)}$, $U^{(k)}$ and $\varphi_{\rm z}$ are all $f_{\rm b}$-dependent. The eigenstates can be solved for a given order of expansion. In general, higher-power expansion terms introduce anharmonicity as well as modulate the harmonic frequency. For simplicity and without loss of generality in the approach, we restrict ourselves to the subsystem spanned by the first-three levels $\{|{\rm g}\rangle, |{\rm e}\rangle, |{\rm f}\rangle\}$. Within a first-order perturbation analysis, we have the approximate Hamiltonian

$$\hat{\mathcal{H}} = \hbar\left(\Omega\hat{b}^\dagger\hat{b} + \frac{\mathcal{A}}{2}\hat{b}^\dagger\hat{b}^\dagger\hat{b}\hat{b}\right) \ , \tag{10}$$

where Ω and $\mathcal{A}$ are respectively the $f_{\rm b}$-dependent harmonicity and anharmonicity. As an important example, they can be derived at $f_{\rm b} = 0$, where $\varphi_{\rm m}^* = 0$. The expansion terms then become

$$\begin{aligned} U^{(2)}/E_{\rm J} &= 1 - 2\alpha \ , \\ U^{(3)}/E_{\rm J} &= 0 \ , \\ U^{(4)}/E_{\rm J} &= \frac{8\alpha - 1}{12} \ , \\ U^{(5)}/E_{\rm J} &= 0 \ , \\ &\ldots \end{aligned} \tag{11}$$

All odd-power terms disappear due to symmetry of the potential at $f_{\rm b} = 0$. The term $U^{(2)}$ provides a quadratic potential and thus harmonicity when $\alpha < 0.5$, which is consistent with the "slightly anharmonic oscillator" model developed here. When $\alpha > 0.5$, as in the conventional flux qubit, the potential becomes a concave function with a rising barrier which splits the landscape into double wells. The quartic potential from $U^{(4)}$ is the leading term that introduces anharmonicity. Keeping terms up to $U^{(4)}$, we have $\hbar\Omega = \sqrt{E_{\rm C,m}E_{\rm J,m}} + \lambda E_{\rm C,m}$ and $\hbar\mathcal{A} = \lambda E_{\rm C,m}$, where $E_{\rm J,m} = 2(1-2\alpha)E_{\rm J}$ and $\lambda = \frac{8\alpha-1}{8(1-2\alpha)}$. Our present C-shunt designs are within the regime of $\alpha > 0.125$, giving a positive anharmonicity in contrast to the negative one which is characteristic in transmon qubits.

Supplementary Note 4.3 C-shunt flux qubit: time-dependent fluctuations

We now consider flux noise and charge noise within this analytical model. Small flux fluctuations (δf) affect this anharmonic system by modulating the potential energy. That is

$$\begin{aligned}\delta\hat{\mathcal{H}}_f = \delta\hat{U} &= -2\pi\alpha E_{\rm J}\sin(2\pi f_{\rm b}+2\varphi_{\rm m})\delta f \\ &= -2\pi\alpha E_{\rm J}\{\sin\phi\,\cos(2\varphi_{\rm m'})+\cos\phi\,\sin(2\varphi_{\rm m'})\}\delta f \\ &\approx -2\pi\alpha E_{\rm J}\{\sin\phi\,(1-2\varphi_{\rm z}^2(\hat{b}+\hat{b}^\dagger)^2)+\cos\phi\,2\varphi_{\rm z}(\hat{b}+\hat{b}^\dagger)\}\delta f\ ,\end{aligned} \tag{12}$$

where $\phi=\phi(f_{\rm b})=2\pi f_{\rm b}+2\varphi_{\rm m}^*(f_{\rm b})$. The $(\hat{b}+\hat{b}^\dagger)^2$ term contains longitudinal modulation while the $(\hat{b}+\hat{b}^\dagger)$ term provides only transverse modulation, that is, a coupling matrix element between adjacent levels.

In the spectral domain, fluctuations near frequency Ω are of particular interest because of their ability to induce $\rm g-e$ transitions, and thus play an essential role in qubit driving and relaxation. Consider a coherent drive $\delta f\propto\cos(\omega_{\rm d}t)$ with driving frequency $\omega_{\rm d}\approx\Omega$. In the interaction frame, one can show that both the fast longitudinal oscillations and transverse counter-rotating terms are negligible in the weak driving limit. This simplifies the three-level interaction-frame Hamiltonian to

$$\hat{\tilde{\mathcal{H}}} = \hbar\begin{pmatrix} 2\Delta\omega+\mathcal{A} & \sqrt{2}\,\Omega_{\rm R}/2 & 0 \\ \sqrt{2}\,\Omega_{\rm R}/2 & \Delta\omega & \Omega_{\rm R}/2 \\ 0 & \Omega_{\rm R}/2 & 0\end{pmatrix}\ , \tag{13}$$

where $\Delta\omega=\Omega-\omega_{\rm d}$ is the detuning of the drive from the $\rm g-e$ transition frequency. In the regime of weak drive, namely $\omega_{\rm q},\mathcal{A}\gg\Omega_{\rm R}$, the system is effectively protected from transitions to the third level. Therefore, we can further reduce the system to two levels,

$$\hat{\mathcal{H}} = \frac{1}{2}\left(\hbar\omega_{\rm q}\,\hat{\sigma}_{\rm z}+I_{\rm m}\Phi_0\,\delta f\,\hat{\sigma}_{\rm x}\right)\ , \tag{14}$$

where $\omega_{\rm q}=\omega_{\rm q}(f_{\rm b})=\Omega$ as in Eq. (10) and $I_{\rm m}=I_{\rm m}(f_{\rm b})=-8\pi\alpha\,\varphi_{\rm z}\cos\phi\,E_{\rm J}/\Phi_0$. Here, $I_{\rm m}$ is the $f_{\rm b}$-dependent current difference between the parameterized circulating-current states (See section Supplementary Note 3). The scaling $I_{\rm m}\propto\alpha E_{\rm J}^{3/4}E_{\rm C}^{1/4}\zeta^{-1/4}$ indicates the efficiency of reducing flux-noise sensitivity by lowering α and $E_{\rm J}$, as we implemented in the C-shunt design. For example, qubit sample B in our device (see main text) has $\alpha\approx0.4$, $E_{\rm J}/h\approx65\,\rm GHz$ and $\varphi_{\rm z}\approx0.28$, leading to $I_{\rm m}\approx55\,\rm nA$.

On the other hand, charge fluctuations ($\delta q_{\rm A}$ and $\delta q_{\rm B}$) invoke perturbation via the kinetic energy $\hat{T}$. Expanding $\hat{T}$ in Eq. (4) and ignoring the p-mode (as before), we have the perturbation Hamiltonian,

$$\begin{aligned}\delta\hat{\mathcal{H}}_q = \delta\hat{T} &= \delta\hat{\mathbf{q}}^{\rm T}\,\mathbf{C}^{-1}\hat{\mathbf{Q}} \\ &= -\frac{e}{C(2\zeta+2\alpha+1)}[-{\rm i}\,n_{\rm z}(\hat{b}-\hat{b}^\dagger)](\delta q_{\rm A}-\delta q_{\rm B})\ .\end{aligned} \tag{15}$$

On the second line, we used the transformation $\hat{n}=-{\rm i}\,n_{\rm z}(\hat{b}-\hat{b}^\dagger)$, where $n_{\rm z}=(E_{\rm J,m}/4E_{\rm C,m})^{1/4}$ is the quantum ground-state uncertainty in Cooper-pair number. Similar to the flux-noise argument above, charge fluctuations also connect to the system transversely, and moreover, are also orthogonal to the flux. In addition, the perturbation depends only on the differential mode of the induced charges between islands A and B, the branch charge across the small junction.

Adding $\delta\hat{\mathcal{H}}_q$ to the two-level approximated Hamiltonian in Eq. (14), we have

$$\hat{\mathcal{H}} = \frac{1}{2}\left(\hbar\omega_{\rm q}\,\hat{\sigma}_{\rm z}+I_{\rm m}\Phi_0\,\delta f\,\hat{\sigma}_{\rm x}+n_{\rm z}E_{\rm C,m}\delta n_{\rm m}\,\hat{\sigma}_{\rm y}\right)\ , \tag{16}$$

where $\delta n_{\rm m}=(\delta q_{\rm A}-\delta q_{\rm B})/(-e)$ is the differential electron number fluctuation. The charge-noise sensitivity is $n_{\rm z}E_{\rm C,m}\propto E_{\rm C,m}^{3/4}\propto\zeta^{-3/4}$. Therefore, the introduction of a large shunt capacitor makes the system less sensitive

to charge fluctuations. In qubit sample B (see main text), $n_{\rm z} E_{\rm C,m}/h \approx 1.2\,$GHz. Assuming Johnson-Nyquist noise from a 377-Ω resistor (free-space impedance) and a parameterized gate capacitance of $0.03\,C = 0.12\,$fF, we find a charge-noise-limited T_1 of about 60 μs.

The capacitive coupling of the qubit loop to the resonator can be modelled in a similar way as the node charges. The voltage fluctuations on the coupling capacitor $C_{\rm g}$ induce charge fluctuations, and hence enter the Hamiltonian through the same channel as charge. Consequently, the interaction Hamiltonian between the qubit and resonator can be written as

$$\hat{\mathcal{H}}_{\rm q-r} = \hbar g\,\hat{\sigma}_{\rm y}\,(\hat{a}^\dagger + \hat{a})\;, \tag{17}$$

where g is the coupling strength.

Supplementary Note 5 Simulation of the Full System Hamiltonian

Supplementary figure 5a shows a schematic of the configuration for the devices described in this work, where the five islands labelled numerically in white text (1-5) follow the electrostatic description of Ref. [1] with island 4 the center conductor of the coplanar waveguide resonator. Supplementary figure 5b shows an equivalent circuit in which islands 1 and 5 have been grounded (equivalent to neglecting the parasitic stripline modes of the resonator). Filled black circles in this circuit indicate the five canonical node variables that would be required for its full quantum description. Junction capacitances are labelled $C_{\rm Ja}$ and $C_{\rm Jb}$ for the small and large junctions, respectively, and $L_{\rm g}$ is the geometric inductance of the qubit loop. Supplementary figures 5c and 5d show successive (approximate) reductions of the circuit to four and three node variables (labelled in blue), respectively. Bold lines in these schematics indicate the chosen spanning tree (equivalent to a choice of gauge) [7, 8].

The quantities in panels (b) and (c) are related to those in (b) according to

$$\begin{aligned} C_{\rm T} &= C_{13} + C_{35} + \frac{C_{34}}{C_4^{\rm tot}}(C_{14} + C_{15}) \\ C_{\rm a} &= C_{23} + \frac{C_{24}}{C_4^{\rm tot}} C_{34} \\ C_{\rm b} &= C_{12} + C_{25} + \frac{C_{24}}{C_4^{\rm tot}}(C_{14} + C_{15}) \\ C_{\rm sh} &= C_{\rm A} + \frac{C_{\rm T} C_{\rm B}}{C_{\rm T} + C_{\rm B}} \end{aligned} \tag{18}$$

where $C_4^{\rm tot} \equiv C_{14} + C_{24} + C_{34} + C_{45}$. The dimensionless capacitive division factor by which the resonator voltage is coupled to the qubit is given by:

$$\begin{aligned} \beta_{\rm c} \;=\;& [2C_{34}(C_{12} + C_{25}) - 2C_{24}(C_{13} + C_{35})] \\ \times\;& \big\{2C_{24}C_{34} + (C_{24} + C_{34})[2(C_{23} + C_{\rm Ja}) + C_{\rm Jb}] + (C_{13} + C_{35})[2(C_{23} + C_{24} + C_{\rm Ja}) + C_{\rm Jb}] \\ & + (C_{12} + C_{25})[2(C_{13} + C_{23} + C_{34} + C_{35} + C_{\rm Ja}) + C_{\rm Jb}]\big\}^{-1}. \end{aligned} \tag{19}$$

Note that the junction capacitances influence this value, in particular when the shunt capacitors are smaller.

Panel (c) is the minimal circuit that fully captures the experimental qubit devices (excluding the resonator), in particular because in nearly all devices the total $C_{\rm sh}$ is determined by the combination of $C_{\rm T}$, $C_{\rm B}$, and $C_{\rm a}$. The approximation inherent in (d) arises from the fact that the two fundamental Josephson-like modes of the circuit (the qubit mode in which nodes 1 and 2 oscillate out of phase, and the "plasma" mode in which they oscillate in

phase) couple to the same shunt capacitance $C_{\rm sh}$. By contrast, in the circuit of panel (c), the plasma mode may couple to a larger capacitance $C_{\rm B} + C_{\rm T} > C_{\rm a}$ than the qubit mode. The result is that, unlike (c) where increasing the shunt capacitance leaves the plasma mode frequency far above the region of interest, in the more realistic circuit of (d) the plasma mode frequency shifts down as the total shunt capacitance is increased, due to the effect of $C_{\rm B}$ and $C_{\rm T}$. For the purpose of the present work, however, it turns out that the circuit of panel (d) gives a good approximation (5% or better) to all quantities considered, except inelastic quasiparticle tunneling matrix elements, where a correct treatment of tunneling events through the two larger junctions precludes grounding one side of those junctions. Note that it is necessary to include the geometric loop inductance (though its effect on the energy levels is small) to account for the flux noise matrix elements to this accuracy.

The Hamiltonians for the two circuits are

$$\begin{aligned}\hat{\mathcal{H}}_3 &= 4\hat{\mathbf{n}}\cdot\mathbf{E}_{\rm C3}\cdot\hat{\mathbf{n}} + E_{{\rm J}\alpha}\big[1-\cos(\hat{\varphi}_2-\hat{\varphi}_1+\varphi_{\rm e})\big] \\ &\quad + E_{\rm Jb}\big[2-\cos(\hat{\varphi}_2-\hat{\varphi}_3)-\cos(\hat{\varphi}_1)\big] \qquad (20)\\ \hat{\mathcal{H}}_4 &= 4\hat{\mathbf{n}}\cdot\mathbf{E}_{\rm C4}\cdot\hat{\mathbf{n}} + E_{{\rm J}\alpha}\big[1-\cos(\hat{\varphi}_2-\hat{\varphi}_1+\varphi_{\rm e})\big] \\ &\quad + E_{\rm Jb}\big[2-\cos(\hat{\varphi}_2-\hat{\varphi}_3)-\cos(\hat{\varphi}_4-\hat{\varphi}_1)\big] + \frac{E_{\rm L}}{2}(\hat{\varphi}_3-\hat{\varphi}_4)^2 \qquad (21)\end{aligned}$$

where $\hat{\mathbf{n}} \equiv \{\hat{n}_i\}$ is the vector node charge operator, $\mathbf{E}_{\rm C3,4} \equiv e^2/2\cdot\mathbf{C}_{3,4}^{-1}$ is the inverse charging energy matrix, $E_{{\rm J}\alpha}$ and $E_{\rm Jb}$ are the Josephson energies of the small and large junctions, respectively, $\hat{\varphi}_i$ are the node phase operators satifying $\big[\hat{\varphi}_j, \hat{n}_k\big] = i\delta_{jk}$, and $\varphi_{\rm e} \equiv 2\pi\Phi_{\rm e}/\Phi_0$ is the dimensionless external flux through the qubit loop. Finally, $E_{\rm L} \equiv (\Phi_0/2\pi)^2/L_{\rm g}$ is the characteristic inductive energy scale for the geometric loop inductance $L_{\rm g}$. The capacitance matrices for the two circuits are given by:

$$\mathbf{C}_3 = \begin{pmatrix} C_{\rm Jb}+C_{\rm Ja}+C_{\rm sh} & -C_{\rm Ja}-C_{\rm sh} & 0 \\ -C_{\rm Ja}-C_{\rm sh} & C_{\rm Jb}+C_{\rm Ja}+C_{\rm sh} & -C_{\rm Jb} \\ 0 & -C_{\rm Jb} & C_{\rm Jb} \end{pmatrix} \qquad (22)$$

$$\mathbf{C}_4 = \begin{pmatrix} C_{\rm B}+C_{\rm Jb}+C_{\rm Ja}+C_{\rm a} & -C_{\rm Ja}-C_{\rm a} & 0 & -C_{\rm Jb} \\ -C_{\rm Ja}-C_{\rm a} & C_{\rm T}+C_{\rm Jb}+C_{\rm Ja}+C_{\rm a} & -C_{\rm Jb} & 0 \\ 0 & -C_{\rm Jb} & C_{\rm Jb} & 0 \\ -C_{\rm Jb} & 0 & 0 & C_{\rm Jb} \end{pmatrix} \qquad (23)$$

To diagonalize the full Hamiltonians for the two circuits, we first set the phase across the inductor to zero and diagonalize the resulting Hamiltonian (which has either two or three node variables) in a truncated charge basis containing the states: $-10, -9, ..., 1, 0, 1, ..., 9, 10$ for each island (in units of Cooper pairs), having dimension $21^2 = 441$ or $21^3 = 9261$. We use the resulting set of eigenstates $\Psi_m^{(0)}$ and eigenenergies $E_m^{(0)}$ to re-express the full Hamiltonian in a product state basis: $\Psi_m^{(0)} \otimes |\nu\rangle$ where $|\nu\rangle$ are linear oscillator states resulting from the loop inductance $L_{\rm g}$ and the shunt capacitance across it. The oscillator basis is truncated at $\nu \leq 3$, and the $L_{\rm g} = 0$ qubit basis is truncated at $m \leq 75$ for the three-node circuit, and $m \leq 300$ for the four node circuit. The increased basis size for the latter case is necessary because the four-node circuit does not have an inductance for its common mode (the one in which all islands oscillate together relative to ground), and therefore no potential energy, resulting in simple, charge eigenstates. The resulting Hilbert space dimensions for the two circuits are $3 \times 75 = 225$ and $3 \times 300 = 900$, much smaller than that which would have otherwise been required: $21^2 \times 3 = 1323$ and $21^3 \times 3 = 27783$ states. This method is a useful way to efficiently include linear inductances into Josephson quantum circuits [9].

Once the eigenenergies and eigenstates of the qubit circuits are determined, this system is again truncated (typically at $\sim$10 qubit energy levels) and then coupled to the resonator using the Hamiltonian

$$\hat{\mathcal{H}}_{\rm q-r} = 2e\,(\hat{n}_1 - \hat{n}_2)\cdot\beta_{\rm c}V_{\rm r0}(\hat{a}^\dagger + \hat{a}), \qquad (24)$$

where β_c is obtained from Eq. (19) above, and $V_{r0} = \omega_r\sqrt{2hZ_r}$ is the rms ground state resonator mode voltage for a mode impedance Z_r. A truncated basis of up to ~ 5 photons in the resonator mode is then used to diagonalize the resulting dressed Hamiltonian.

Supplementary Note 6 High-Power Spectrum and Higher-Level Qubit Transitions

To characterize higher levels of the qubit, we perform high-power qubit spectroscopy (Supplementary Figure 6). The measurement consists of scanning the drive frequency (ω_d) as a function of the flux bias with a much higher power than that used in the main text in Fig. 2a. Strong driving reveals more qubit transitions in the spectrum, including the first four qubit levels, single and multi-photon transitions, and resonator-mediated transitions. The same simulation used to develop our noise models is used to add the solid lines in Supplementary Figure 6. To match the measured transition frequencies optimally across the entire flux bias range, we generally need to tune somewhat the design parameters (Supplementary Table 1), typically by $10-25\%$. Once adjusted, the fitting is very good and reproduces the measured spectroscopy over a wide range of flux and frequency values.

To characterize the anharmonicity accurately, we use a two-tone low-power pulse technique to measure 0–1 and 1–2 transitions sequentially (Supplementary Figure 7). A short π-pulse at 0–1 transition frequency first prepares the qubit at $|1\rangle$ state. Then, a low-power frequency scan resolves the 0–1 and 1–2 transitions. The frequency difference gives the anharmonicity which is 490 MHz for Qubit B. This is somewhat less than the 830 MHz predicted solely from the design values (Supplementary Table 1). Although the measured value is off by approximately 330 MHz from the anharmonicity predicted directly from the design values, it is important to keep in mind that this few-hundred MHz difference is on top of qubit frequencies more than 10 times larger (i.e., 4.7-5.2 GHz) and, in this sense, the error is rather small.

Supplementary Note 7 Qubit Parameters

The parameters for the 22 qubits studied in the work are listed in Supplementary Table 1.

Supplementary Note 8 Noise Models

The total T_1 of the qubit is taken to be:

$$\frac{1}{T_1} = \frac{1}{T_1^{\Phi}} + \frac{1}{T_1^{Q}} + \frac{1}{T_1^{\mathrm{qp}}} + \frac{1}{T_1^{\mathrm{P}}} \tag{25}$$

where the terms come from electric noise, magnetic noise, inelastic quasiparticle fluctuations, and the Purcell effect, respectively.

The coupling of electric and magnetic noise to the qubit is modeled as shown in Supplementary Figure 8, in terms of voltage sources δV_i weakly coupled to the circuit islands, and a current source δI_l weakly coupled to the circuit loop. We assume the weak-coupling limit for both of these, where C_{ci} are negligible compared to the corresponding node capacitances (diagonal elements of $\mathbf{C}$), and M_c is negligible compared to the qubit's loop

inductance L_g. In this limit, the electric noise can be expressed as well-defined charge fluctuations δQ_i for node i (with magnitudes independent of the node capacitances), and the magnetic noise as flux fluctuation through the loop $\delta\Phi_l$ (with magnitude independent of the loop inductance). The coupling Hamiltonians for these fluctuations to the qubit can be written:

$$\hat{\mathcal{H}}_{\delta\Phi} = \hat{I}_l \delta\Phi_l \quad (26)$$

$$\hat{\mathcal{H}}_{\delta Q_i} = \hat{V}_i \delta Q_i \quad (27)$$

where $\hat{I}_l \equiv \Phi_0(\hat{\varphi}_3 - \hat{\varphi}_4)/2\pi/L_\mathrm{g}$ is the loop current operator and $\hat{V}_i \equiv (\hat{\mathbf{Q}} \cdot \mathbf{C}^{-1})_i$ is the voltage operator for node i. The resulting contributions to the total decay rate are:

$$\frac{1}{T_1^\Phi} = \frac{1}{\hbar^2} \left|\langle \mathrm{e}|\hat{I}_l|\mathrm{g}\rangle\right|^2 S_\Phi(\omega_\mathrm{q}) \quad (28)$$

$$\frac{1}{T_1^Q} = \frac{1}{\hbar^2} \left|\langle \mathrm{e}|\hat{\mathbf{V}}|\mathrm{g}\rangle\right|^2 S_Q(\omega_\mathrm{q}) \quad (29)$$

For the Purcell-enhanced decay rate of the qubit excited state, we use the expression:

$$\frac{1}{T_1^\mathrm{P}} = \frac{1}{\hbar^2} \left|\langle \tilde{\mathrm{e}}_{1\gamma}|\hat{\mathcal{H}}_{\mathrm{q-r}}|\tilde{\mathrm{g}}_{1\gamma}\rangle\right|^2 \kappa \quad (30)$$

where $\hat{\mathcal{H}}_{\mathrm{q-r}}$ is defined in Eq. (17) above, κ is the resonator decay rate, and $|\tilde{\mathrm{g}}_{1\gamma}\rangle$, $|\tilde{\mathrm{e}}_{1\gamma}\rangle$ are the two dressed energy eigenstates of the qubit/resonator system in the one-photon subspace.

As shown in purple in Supplementary Figure 8, the quasiparticle noise contribution to the decay rate is modeled, following Ref. 10, as a parallel admittance Y_qp and corresponding current fluctuations δI. This approximate description is justified since $Y_\mathrm{qp}(\omega_\mathrm{q}) \ll Y_\mathrm{J}(\omega_\mathrm{q})$ where Y_J is the junction impedance. The resulting decay rate is [10, 11]:

$$\frac{1}{T_1^\mathrm{qp}} = \frac{1}{4} \sum_{k=1}^{3} \left|\langle \tilde{\mathrm{e}}_{1k}|\hat{t}^k_\mathrm{qp} e^{i\varphi_k/2} - \hat{t}^{k\dagger}_\mathrm{qp} e^{-i\varphi_k/2}|\tilde{\mathrm{g}}_{1k}\rangle\right|^2 S^k_\mathrm{qp}(\omega_\mathrm{q}) \quad (31)$$

where the sum is over the three junctions, the operator $\hat{t}^k_\mathrm{qp}$ transfers a quasiparticle through junction k, the phase offsets are $\varphi_k = \varphi_\mathrm{e}$ for the small junction and zero otherwise, and $|\tilde{g}_{1k}\rangle$, $|\tilde{e}_{1k}\rangle$ are qubit energy eigenstates in the presence of a single quasiparticle on one electrode of junction k. The effective quasiparticle current noise spectral density S^k_qp for junction k is given approximately by [10, 11]:

$$S^k_\mathrm{qp} = x_\mathrm{qp} \frac{E_{\mathrm{J}k}}{h} \sqrt{\frac{8\Delta_\mathrm{Al}}{\omega_\mathrm{q}}} \quad (32)$$

where x_qp is the dimensionless quasiparticle density (scaled by the density of superconducting electrons), and Δ_Al is the superconducting energy gap of aluminum. The eigenstates of the qubit in the presence of a single quasiparticle on each circuit node are obtained using a modified charge representation for that node with 20 basis states: $-19/2, -17/2, ..., -1/2, 1/2, ..., 17/2, 19/2$ (in units of Cooper pairs). The Hamiltonian is separately diagonalized for each single-quasiparticle configuration using the methods described above. Then the matrix elements in Eq. (31) above are evaluated between eigenstates of these different configurations, corresponding to a single quasiparticle moving through each of the three Josephson junctions in the loop. The resulting decay rates associated with inelastic tunneling through each junction are summed to produce the total rate. Note that we neglect processes associated with the presence of two quasiparticles simultaneously, which is justified based on our observation of $\bar{n}_\mathrm{qp} \approx 0.26$.

Supplementary Note 9 Definitions of Power Spectral Densities

The spectral data shown in Fig. 3b in the main text represent a symmetrized PSD, the Fourier transform of the symmetrized autocorrelation function,

$$S(\omega) = \int_{-\infty}^{\infty} \mathrm{d}\tau \, \exp(-i\omega\tau) \, \frac{1}{2}\langle \hat{\lambda}(0)\hat{\lambda}(\tau) + \hat{\lambda}(\tau)\hat{\lambda}(0) \rangle \, , \tag{33}$$

where $\hat{\lambda}$ is the correlator of interest and is an operator. Clearly, by definition, $S(-f) = S(f)$, and the effects of classical and quantum noise are represented equally at both positive and negative frequencies.

In comparison, the unsymmetrized definition for the PSD,

$$S^{\mathrm{U}}(\omega) = \int_{-\infty}^{\infty} \mathrm{d}\tau \, \exp(-i\omega\tau) \, \langle \hat{\lambda}(0)\hat{\lambda}(\tau) \rangle \, , \tag{34}$$

explicitly distinguishes absorption and emission (stimulated and spontaneous) by the qubit. The emission and absorption rates are related to the positive and negative part of the spectrum respectively,

$$\begin{aligned} \Gamma_{-} &= \frac{1}{4} S^{\mathrm{U}}(\omega_{\mathrm{q}}) \, , \\ \Gamma_{+} &= \frac{1}{4} S^{\mathrm{U}}(-\omega_{\mathrm{q}}) \, . \end{aligned} \tag{35}$$

At equilibrium temperature,

$$\frac{S^{\mathrm{U}}(-\omega_{\mathrm{q}})}{S^{\mathrm{U}}(\omega_{\mathrm{q}})} = \exp\left(-\frac{\hbar\omega}{k_{\mathrm{B}}T}\right) \, . \tag{36}$$

This equation indicates that, in the classical, low-frequency limit ($\hbar\omega \ll k_{\mathrm{B}}T$), $S(\omega) = S^{\mathrm{U}}(\omega) = S^{\mathrm{U}}(-\omega)$. In the quantum, high-frequency limit ($\hbar\omega \gg k_{\mathrm{B}}T$), $S^{\mathrm{U}}(\omega) = 2S(\omega)$. Therefore, a factor 2 difference arises at high frequencies between the two PSD definitions. Since the $1/f$ noise at low frequencies is classical, arising from an ensemble of fluctuators [12], we elect to use the symmetrized PSD. Then, to make a meaningful comparison between the $1/f$ noise PSD and the corresponding values from the measured and simulated emission rates of the qubit, we must be cognizant of these two definitions [13] in order to plot the data appropriately.

Supplementary Note 10 Noise Spectroscopy via Spin Locking

Supplementary Note 10.1 Spin locking technique

Spin-locking or $T_{1\rho}$ noise spectroscopy is an accurate method developed for resolving noise power spectral densities (PSD) by measuring qubit relaxation rates in the rotating frame during driven evolution. The spectroscopy spans the intermediate frequency range, *i.e.,* achievable Rabi frequencies, without substantially undermining the locking condition. Details of this method are discussed in Ref. [14].

When a two-level system (TLS) is driven by a weak ($\omega_{\mathrm{R}} \ll \omega_{\mathrm{q}}$) and resonant ($\omega_{\mathrm{R}} \gg \Delta\omega$) tone, evolution can be conveniently described in the rotating frame, which revolves around the z-axis at the drive frequency (Supplementary Figure 9a). It can be viewed as a fictitious TLS with a quantizing field pointing to X. The level splitting is now the (locking) Rabi frequency, ω_{R}, rather than ω_{q} for the free-evolution case. Note that the corresponding longitudinal relaxation time, $T_{1\rho}$, is defined with respect to the new quantization axis. The source of the relaxation

is noise at the Rabi frequency, transverse to the X-axis. Given the definition in Ref. [14], the rate $\Gamma_{1\rho}$ can be expressed as

$$\begin{aligned}\Gamma_{1\rho} = \frac{1}{T_{1\rho}} &= \frac{1}{2}S_{\perp\mathrm{X}}(\omega_\mathrm{R}) \\ &= \frac{1}{2}\big[S_\mathrm{Y}(\omega_\mathrm{R}) + S_\mathrm{Z}(\omega_\mathrm{R})\big] \\ &= \frac{1}{2}\big[\frac{1}{4}S_\mathrm{x}(\omega_\mathrm{q}+\omega_\mathrm{R}) + \frac{1}{4}S_\mathrm{x}(\omega_\mathrm{q}-\omega_\mathrm{R})\big] + \frac{1}{2}S_\mathrm{z}(\omega_\mathrm{R}) \\ &\approx \frac{1}{4}S_\mathrm{x}(\omega_\mathrm{q}) + \frac{1}{2}S_\mathrm{z}(\omega_\mathrm{R}) \\ &= \frac{1}{2}\Gamma_1 + \Gamma_\nu \ . \end{aligned} \tag{37}$$

Here, $\Gamma_1 = 1/T_1 = \frac{1}{2}S_\mathrm{x}(\omega_\mathrm{q})$ is the qubit-frame longitudinal relaxation rate and $\Gamma_\nu = \frac{1}{2}S_\mathrm{Z}(\omega_\mathrm{R})$ is the rate associated with the Rabi-frequency noise. In Eq. (37), the contributing rotating-frame noise in the second line is transformed to qubit-frame noise in the third line. The "1/4"-factor arises from the halved noise amplitude at the positive sideband.

Equation (37) suggests that the noise PSD at the Rabi frequency can be extracted by measuring the qubit-frame and rotating-frame longitudinal relaxation rates and making the appropriate subtraction. The spin-locking technique [15–17] is a straightforward way to measure the rotating-frame longitudinal relaxation. We use the standard three-pulse spin-locking sequence (Supplementary Figure 9b), under which the qubit undergoes $T_{1\rho}$ relaxation during the continuous driving pulse (Supplementary Figure 9c). The recorded decay is fit to an exponential function to derive the damping rate $\Gamma_{1\rho}$.

Supplementary Note 10.2 Spectral density for thermal photons in a resonator

Thermal photons in a resonator with decay rate κ have an exponential two-time photon-number autocorrelation function $C(\tau)$ in the small $\bar{n}$ limit [18, 19],

$$C(\tau) = \bar{n}\exp(-\kappa\tau). \tag{38}$$

Correspondingly, by the Weiner-Khinchin theorem, the associated power spectral density (PSD) of the thermal photons in the resonator is the fourier transform of Eq. (38),

$$S_{nn}(\omega) = \frac{2\kappa\bar{n}}{\omega^2+\kappa^2}. \tag{39}$$

From Bloch-Redfield theory, longitudinal relaxation is connected with the two-time correlation of the transverse noise. This also applies to the rotating-frame analogue in the $T_{1\rho}$ process. The measured $T_{1\rho}$ noise spectrum is thus related to the traditional PSD. The effect from the non-Gaussian statistics in the photon noise can be ignored in such relaxation process. To derive the effective PSD as seen by the qubit, one must account for the dispersive coupling χ of the qubit to the resonator and its associated Stark shift (i.e., the frequency shift per photon), and the factor $\eta = \kappa^2/(\kappa^2+4\chi^2)$ that scales the effective photon population seen by the qubit. The resulting PSD is

$$S_\mathrm{z}(\omega) = (2\chi)^2\,[S_{nn}(\omega)]_{\bar{n}\to\eta\bar{n}} = (2\chi)^2\frac{2\kappa\eta\bar{n}}{\omega^2+\kappa^2}. \tag{40}$$

Supplementary Note 11 Comparing Models for Ohmic Charge Noise and Ohmic Flux Noise

In the main text Fig. 3a (device C), it is clear that our data clearly support the position that $1/f$ flux noise is a T_1 mechanism. However, above 3 GHz in this device, there is an ambiguity between ohmic charge noise (magenta dahsed line, Fig. 3a) and ohmic flux noise (grey dashed line, Fig. 3a). We used ohmic charge noise in our models in the main text, because there is a known physical basis for its role in relaxation from prior work and it gave a slightly better match to experimental results across all 22 devices. In Supplementary Figures 10a - 10f, we apply our model to the flux dependence of devices B and C, and to the prediction of T_1 at the flux insensitive points of all 22 qubits under two conditions:

- using ohmic charge noise as was done in the main text,
- using ohmic flux noise in place of ohmic charge noise.

We note that the two models give a similarly reasonable match to the data for all qubits. The one possible exception is for device C (Supplementary Figures 10a and 10b) above 6 GHz, where this device is highly sensitive to charge noise. In this region, one might make a plausible distinction between the efficacy of the two models. Making a stronger distinction between ohmic charge noise and ohmic flux noise will be a topic of future work.

Supplementary Note 12 Thermal Photon Noise in the Low-Number Regime

The long-time behavior in Stark shift ($\Delta^{\mathrm{th}}_{\mathrm{Stark}}$) and dephasing rate ($\Gamma^{\mathrm{th}}_{\varphi}$) due to thermal photons has a nonlinear dependence on the photon number ($\bar{n}$) in general. Equations (43)-(44) in Ref. [20] give the dependence for an arbitrary ratio between χ and κ,

$$\begin{aligned} \Delta^{\mathrm{th}}_{\mathrm{Stark}} &= \frac{\kappa}{2}\,\mathrm{Im}[\sqrt{Z}\,] - \chi \,, \\ \Gamma^{\mathrm{th}}_{\varphi} &= \frac{\kappa}{2}\,\mathrm{Re}\,[\sqrt{Z}\,] - \frac{\kappa}{2} \,, \end{aligned} \tag{41}$$

where $Z = (1 + i\,2\chi/\kappa)^2 + i\,8\chi/\kappa$. Solving for $\sqrt{Z}$, we have

$$\begin{aligned} \mathrm{Im}[\sqrt{Z}\,] &= \sqrt{\frac{-(1-r^2) + \sqrt{(1+r^2)^2 + 16r^2\bar{n} + 16r^2\bar{n}^2}}{2}} \,, \\ \mathrm{Re}[\sqrt{Z}\,] &= \sqrt{\frac{(1-r^2) + \sqrt{(1+r^2)^2 + 16r^2\bar{n} + 16r^2\bar{n}^2}}{2}} \,, \end{aligned} \tag{42}$$

where $r = 2\chi/\kappa$.

In this work, we are focusing on the situation when $\bar{n}$ is much smaller than 1. Expanding Eq. (42) to first order in $\bar{n}$ yields:

$$\begin{aligned} \mathrm{Im}[\sqrt{Z}\,] &= r + \frac{2r}{1+r^2}\bar{n} \,, \\ \mathrm{Re}[\sqrt{Z}\,] &= 1 + \frac{2r^2}{1+r^2}\bar{n} \,. \end{aligned} \tag{43}$$

Substituting these expressions into Eqs. (41) gives Eqs. (4)-(5) in the main text. In fact, the low-number condition is r-dependent. The linear approximation is valid when

$$\begin{aligned}\bar{n} &\ll \frac{(1+r^2)^2}{16r^2} \\ &= \frac{1}{16}(2+r^2+\frac{1}{r^2}) \; .\end{aligned} \tag{44}$$

Therefore, the condition becomes much looser when the system is in either the strong ($r > 1$) or weak coupling ($r < 1$) regime, meaning that the linear dependence can extend to higher photon number in both cases. The condition is tightest ($\bar{n} \ll 1/4$) when $r = 1$.

Supplementary Note 13 CPMG pulse sequence and filter functions

The Carr-Purcell [21]-Meiboom-Gill [22] (CPMG) is a dynamical decoupling pulse sequence that is the multi-pulse generalization of the Hahn spin-echo [23]. The CPMG sequence comprises equally spaced π-pulses in quadrature (with phases 90°-shifted) with respect to the initial $\pi/2$-pulse. The technique reduces dephasing due to low-frequency noise by a coherent refocusing effect imparted by the π-pulses. The act of applying π-pulses in the time-domain can be treated in the frequency domain as a band-pass filter that shapes the noise spectra. The passband of this filter is inversely related to the spacing between adjacent π-pulses.

During free evolution, the decay function due to dephasing is written $\exp[-\xi(\tau)]$, where $\xi(\tau)$ is called the coherence function and τ is the total free-evolution time. Assuming a Gaussian noise environment, the coherence function is

$$\xi(\tau) = \tau^2 \int_0^\infty \frac{\mathrm{d}\omega}{2\pi}\, S_\mathrm{z}(\omega)\, F(\omega,\tau), \tag{45}$$

where $S_\mathrm{z}(\omega)$ is the power spectral density of the longitudinal noise that causes the dephasing, and $F(\omega,\tau)$ is a sequence-specific weighting function called the filter function which acts to shape the noise spectrum seen by the qubit [24]. Assuming infinitely short pulses, the filter function for a CPMG sequence with N (even) π-pulses is $F_\mathrm{CP}^{(N)}(\omega,\tau) = 4\,\mathrm{sinc}^2(\omega\tau/2)\sin^4(\omega\tau/4N)/\cos^2(\omega\tau/2N)$. As illustrated in Supplementary Figure 11, this filter is essentially a bandbpass filter with a passband that peaks around frequency $\omega/2\pi = N/2\tau$, indicating that more π-pulses will shift the filter to higher frequencies. In addition, the filter bandwidth for a fixed passband becomes narrower with larger N.

Taking into account the effect from finite duration of π-pulses, the modified filter function has a general form [25, 26]

$$F_\mathrm{CP}^{(N)}(\omega,\tau) = \frac{1}{(\omega\tau)^2}\left|1+(-1)^{1+N}\exp(\mathrm{i}\,\omega\tau) + 2\sum_{j=1}^{N}(-1)^j \exp(\mathrm{i}\,\omega\delta_j\tau)\cos(\omega\tau_\pi/2)\right|^2, \tag{46}$$

where $\delta_j \in [0,1]$ is the normalized position of the center of the jth π-pulse between the two $\pi/2$-pulses and τ_π is the length of each π-pulse, yielding a total sequence length $\tau + N\tau_\pi$. For π-pulses of short duration (*e.g.,* $\tau_\pi = 10$ ns) compared with the total free-evolution time (*i.e.,* $N\tau_\pi \ll \tau$), as is typical for our experiment, the bandpass filter frequency still peaks near $\omega/2\pi = N/2\tau$. In practice, we use Eq. (46) to find its precise position.

The photon shot-noise measured in this work has a Lorentzian noise power spectral density centered at zero-frequency. The spectral density is essentially frequency independent at low frequencies (the "white-noise" region of the Lorenzian), and it decreases at higher frequencies (the "tail" of the Lorenzian). For small N, such that the filter passband is in the white-noise region, the dephasing time T_ϕ does not change with N. For large enough N,

such that the filter passband reaches the Lorentzian tail region, the noise power contributing to the coherence integral in Eq. (45) is reduced. For such large N, the dephasing time T_ϕ increases as N increases. Since the transverse relaxation time is defined (within a Bloch-Redfield picture) by the rates $1/T_2 = 1/2T_1 + 1/T_\phi$, increasing N in this tail region will extend T_2 towards the $2T_1$ limit.

For the qubit described in the main text, $T_{2,\mathrm{CPMG}}$ is approximately $40\,\mu$s for $N \leq 100$ and represents a typical duration τ of the free-evolution. Furthermore, as seen in Fig. 6b in the main text, $T_{2,\mathrm{CPMG}}$ begins to increase around $N = 100$, reaching $T_{2,\mathrm{CPMG}} = 50\,\mu$s at $N = 200$. Taking $\tau = 50\,\mu$s and $N = 200$, the characteristic frequency is $200/(2 \times 50\,\mu\mathrm{s}) = 2\,$MHz, consistent with the -3dB point of the Lorentzian spectrum with bandwidth $\kappa/2\pi = 1.5\,$MHz (see Fig. 6a in the main text). For $N > 1000$, $T_{2,\mathrm{CPMG}}$ saturates at about $85\,\mu$s, close to the expected value of $2T_1$.

References

[1] Koch, J. *et al.* Charge-insensitive qubit design derived from the cooper pair box. *Phys. Rev. A* **76**, 042319 (2007).

[2] Dolan, G. Offset masks for liftoff photoprocessing. *Appl. Phys. Lett.* **31**, 337 (1977).

[3] Orlando, T. P. *et al.* Superconducting persistent-current qubit. *Phys. Rev. B* **60**, 15398–15413 (1999). URL `http://link.aps.org/doi/10.1103/PhysRevB.60.15398`.

[4] Berns, D. M. *et al.* Amplitude spectroscopy of a solid-state artificial atom. *Nature* **455**, 51 (2008). URL `http://dx.doi.org/10.1038/nature07262`.

[5] Valenzuela, S. O. *et al.* Microwave-induced cooling of a superconducting qubit. *Science* **314**, 1589–1592 (2006). URL `http://www.sciencemag.org/content/314/5805/1589.abstract`. `http://www.sciencemag.org/content/314/5805/1589.full.pdf`.

[6] Girvin, S. Circuit qed: Superconducting qubits coupled to microwave photons. *in Proceedings of the 2011 Les Houches Summer School* (2011).

[7] Devoret, M. Quantum fluctuations in electrical circuits. *Quantum Fluctuations, Les Hourches Session LXIII* (1997).

[8] Yurke, B. & Denker, J. Quantum network theory. *Phys. Rev. A* **29**, 1419–1437 (1984).

[9] To be published.

[10] Catelani, G. *et al.* Quasiparticle relaxation of superconducting qubits in the presence of flux. *Phys. Rev. Lett.* **106**, 077002 (2011). URL `http://link.aps.org/doi/10.1103/PhysRevLett.106.077002`.

[11] Wenner, J. *et al.* Excitation of superconducting qubits from hot nonequilibrium quasiparticles. *Phys. Rev. Lett.* **110**, 150502 (2013).

[12] Schriefl, J., Makhlin, Y., Shnirman, A. & Schn, G. Decoherence from ensembles of two-level fluctuators. *New Journal of Physics* **8**, 1 (2006). URL `http://stacks.iop.org/1367-2630/8/i=1/a=001`.

[13] Shnirman, A., Schön, G., Martin, I. & Makhlin, Y. Low- and high-frequency noise from coherent two-level systems. *Phys. Rev. Lett.* **94**, 127002 (2005). URL `http://link.aps.org/doi/10.1103/PhysRevLett.94.127002`.

[14] Yan, F. *et al.* Rotating-frame relaxation $T_{1\rho}$ as a noise spectrum analyzer of a superconducting qubit undergoing driven evolution. *Nature Commun.* **4** (2013). URL `http://dx.doi.org/10.1038/ncomms3337`.

[15] Slichter, C. P. & Ailion, D. Low-field relaxation and the study of ultraslow atomic motions by magnetic resonance. *Phys. Rev.* **135**, A1099–A1110 (1964). URL `http://link.aps.org/doi/10.1103/PhysRev.135.A1099`.

[16] Ailion, D. C. & Slichter, C. P. Observation of ultra-slow translational diffusion in metallic lithium by magnetic resonance. *Phys. Rev.* **137**, A235–A245 (1965). URL `http://link.aps.org/doi/10.1103/PhysRev.137.A235`.

[17] Look, D. C. & Lowe, I. J. Nuclear magnetic dipole—dipole relaxation along the static and rotating magnetic fields: Application to gypsum. *The Journal of Chemical Physics* **44**, 2995–3000 (1966). URL `http://link.aip.org/link/?JCP/44/2995/1`.

[18] Mandel, L. & Wolf, E. *Optical Coherence and Quantum Optics* (Cambridge University Press, 1995).

[19] Bertet, P., Chiorescu, I., Harmans, C. & Mooij, J. Dephasing of a flux-qubit coupled to a harmonic oscillator. Preprint at http:/arxiv.org/abs/cond-mat/0507290 (2005).

[20] Clerk, A. A. & Utami, D. W. Using a qubit to measure photon-number statistics of a driven thermal oscillator. *Phys. Rev. A* **75**, 042302 (2007). URL `http://link.aps.org/doi/10.1103/PhysRevA.75.042302`.

[21] Carr, H. Y. & Purcell, E. M. Effects of diffusion on free precession in nuclear magnetic resonance experiments. *Phys. Rev.* **94**, 630–638 (1954). URL `http://link.aps.org/doi/10.1103/PhysRev.94.630`.

[22] Meiboom, S. & Gill, D. Modified spin echo method for measuring nuclear relaxation times. *Review of Scientific Instruments* **29**, 688–691 (1958).

[23] Hahn, E. L. Spin echoes. *Phys. Rev.* **80**, 580–594 (1950). URL `http://link.aps.org/doi/10.1103/PhysRev.80.580`.

[24] Cywiński, L., Lutchyn, R. M., Nave, C. P. & Das Sarma, S. How to enhance dephasing time in superconducting qubits. *Phys. Rev. B* **77**, 174509 (2008). URL `http://link.aps.org/doi/10.1103/PhysRevB.77.174509`.

[25] Biercuk, M. J. *et al.* Optimized dynamical decoupling in a model quantum memory. *Nature* **458**, 996–1000 (2009). URL `http://dx.doi.org/10.1038/nature07951`.

[26] Biercuk, M. J. *et al.* Experimental uhrig dynamical decoupling using trapped ions. *Phys. Rev. A* **79**, 062324 (2009). URL `http://link.aps.org/doi/10.1103/PhysRevA.79.062324`.
